# Kir7.1 is the physiological target for hormones and steroids that regulate uteroplacental function

**DOI:** 10.1126/sciadv.adr5086

**Published:** 2025-03-05

**Authors:** Monika Haoui, Citlalli Vergara, Lina Kenzler, Jerome Schröer, Geraldine Zimmer-Bensch, David Fleck, Christopher Wiesbrock, Marc Spehr, Polina V. Lishko

**Affiliations:** ^1^ Department of Molecular and Cell Biology, University of California, Berkeley, CA, USA.; ^2^ Center for the Investigation of Membrane Excitability Diseases (CIMED), WashU Medicine; Washington University in St. Louis, School of Medicine, St. Louis, MO, USA.; ^3^ Department of Chemosensation, Institute for Biology II, RWTH Aachen University, 52074 Aachen, Germany.; ^4^ Department of Neuroepigenetics, Institute for Biology II, RWTH Aachen University, 52074 Aachen, Germany.

## Abstract

Preterm birth is detrimental to the well-being of both the mother and the newborn. During normal gestation, the myometrium is maintained in a quiescent state by progesterone. As a steroid hormone, progesterone is thought to modify uterine and placental morphology by altering gene expression, but another direct mode of action has long been suspected. Here, we reveal the nongenomic molecular mechanism of progesterone as the activation of human and murine inwardly rectifying potassium channel Kir7.1, which is expressed in myometrium and placental pericytes during late gestation. Kir7.1 is also activated by selective steroids, including those used to prevent premature labor, such as 17-α-hydroxyprogesterone caproate and dydrogesterone, revealing their unexpected mode of action. Our results reveal that Kir7.1 is the molecular target of both endogenous and synthetic steroids that control uterine excitability and placental function. Kir7.1, therefore, is a promising therapeutic target to support healthy pregnancy during mid and late gestation.

## Teaser

Progesterone and progestins activate Kir7.1 to control uterine excitability, offering insights for preterm birth therapy.

## INTRODUCTION

The uterus is among the strongest smooth muscles in the body per mass of tissue (*1*). During parturition, the uterus produces strong and synchronized myometrial contractions, ensuring successful delivery. However, during pregnancy, the uterus must remain quiescent to support the developing fetus and prevent premature labor (*2*, *3*). Uterine contractility is, therefore, controlled by endocrine factors, including the steroid hormone progesterone (P4), making it a steroid hormone–responsive organ (*4*–*6*). In humans, most of P4 that is generated during pregnancy is produced by the placenta (*7*), a heavily vascularized organ that also supports embryogenesis and provides nutrient exchange between the mother and the fetus (*8*). “P4 block,” the theory proposed in 1956 (*9*), has been recognized as the main factor supporting pregnancy and gestational uterine quiescence. High P4 levels in blood circulation are retained throughout pregnancy and maintain myometrial quiescence, preventing premature delivery (*5*). However, the pleiotropic molecular mechanism underlying P4 action on the myometrium is not completely understood (*6*, *10*–*13*). In most mammals, a drop in P4 levels, known as systemic P4 withdrawal, is required for myometrial excitation and consequently, the onset of labor (*5*, *14*, *15*). This differs in primates during parturition, where high P4 levels are retained, and, yet, primate myometrium is unable to maintain quiescence (*6*). This phenomenon has been described as “functional withdrawal” and is now incompletely understood (*5*, *10*, *12*). Altered activity of the nuclear progesterone receptor only partially explains both effects, i.e., P4-dependent maintenance of myometrial quiescence throughout most of the pregnancy, as well as its functional withdrawal in primates (*10*, *12*, *16*). Therefore, non-nuclear targets of P4 that modify cellular excitability, such as ion channels, have been long suspected (*17*). This hypothesis is supported by reports of rapid and reversible uterine relaxation upon acute application of P4 to isolated myometrial strips (*11*, *18*).

Here, we show that uterine contractility is controlled by P4 via inwardly rectifying potassium channel Kir7.1. This channel is expressed specifically at the site of implantation in the myometrium. Functional Kir7.1 uterine expression is restricted to late gestation, and, moreover, previous reports showed that Kir7.1 knockdown increases uterine contractility, while reintroducing Kir7.1 after knockdown restores uterine quiescence (*17*). However, the endogenous regulators of this channel were not known. By combining electrophysiological recordings from recombinantly expressed Kir7.1, as well as from isolated uterine myocytes with in situ recording from murine uterine tissues at late gestation, we report that both murine and human Kir7.1 are activated by P4 via a non-genomic mechanism. In vitro activation of human Kir7.1 by P4 was antagonized by 17β-estradiol (E2). The latter has been previously shown to rise before parturition in most mammals (*14*). The inability of P4 to activate Kir7.1 in the presence of E2 and, hence, its weaker influence on myometrial relaxation could explain, at least partially, the phenomenon of functional withdrawal. Steroid-sensitivity profile of Kir7.1 was comprehensively evaluated, revealing that the ion channel is only activated by two pregnancy-related hormones, dehydroepiandrosterone (DHEA) and P4, while other endogenous steroids fail to activate Kir7.1. Additionally, we examined a compound used to prevent preterm labor, 17-α-hydroxyprogesterone caproate (17-OHPC), as well as another synthetic steroid, dydrogesterone, which is now in trials to treat preeclampsia (*19*, *20*). Both drugs were, so far, expected to act via nuclear P4 receptor but are potent activators of human Kir7.1. The abortifacient drug mifepristone (RU486), a compound that causes strong myometrial contractions (*21*), was revealed to act as a Kir7.1 antagonist that, at therapeutical concentration, counteracts the effect of P4. Additionally, we revealed functional expression of Kir7.1 in placental pericytes, specific mural cells that enwrap blood capillaries and control the blood supply to the fetus (*8*).

Together, these data reveal a mechanism of action exerted by steroids to maintain pregnancy outcome. Moreover, we provide strong evidence for an unconventional nongenomic mechanism behind the rapid control of uterine excitability and placental physiology by P4.

## RESULTS

### Kir7.1 is functionally expressed in murine myometrial smooth muscle during late gestation

**Fig. 1.**
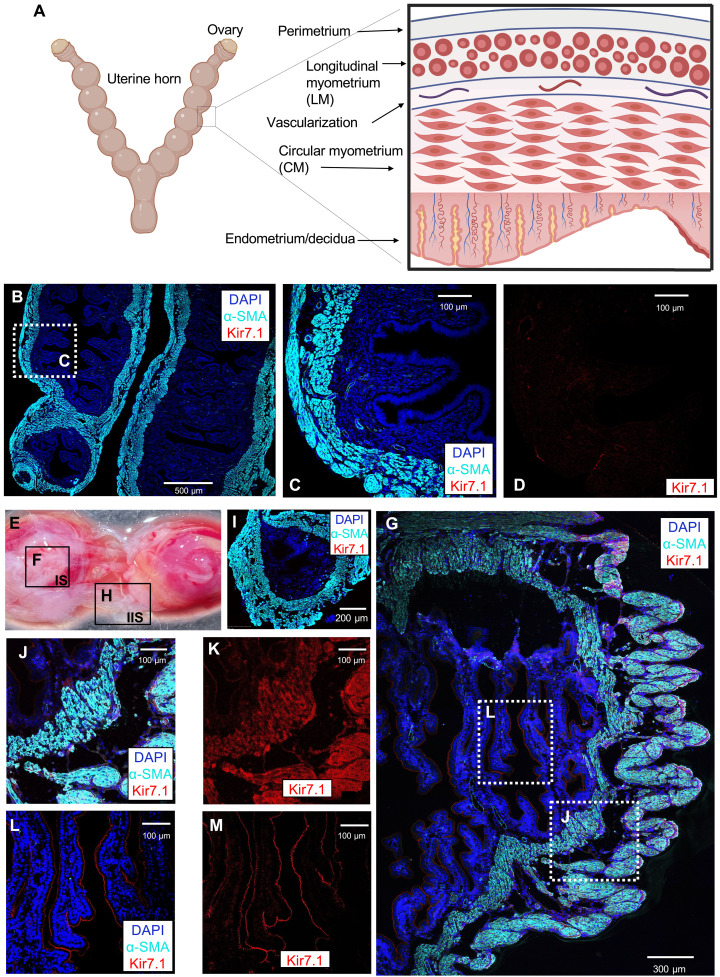
Kir7.1 expression and localization in the murine myometrium. (**A**) Schematic representation of mouse uterine morphology during gestation. The insert shows different uterine layers, including the endometrium, circular myometrial inner layer, vascular and longitudinal myometrial layers. Created with BioRender.com. (**B**) Nonpregnant uterus co-stained with anti–smooth muscle α-actin antibody (α-SMA; cyan), anti-Kir7.1 antibody (red), and 4′,6-diamidino-2-phenylindole (DAPI; blue, indicating nuclei). Lack of red signal indicates absence of Kir7.1 expression. (**C**) Zoomed-in portion of myometrium corresponding to dotted frame in (B) with all three channels superimposed. (**D**) Portion of myometrium (same as in C) with only single channel (anti-Kir7.1 antibody; red) shown, indicating an absence of Kir7.1. (**E**) Isolated mouse uterus at 15.5 days post coitum (dpc) with placentae (red tissue) and myometrium (white tissue) shown. Two separate regions of myometrium: adjacent to placenta [(**F**); IS, implantation site] and between placentae [(**H**); IIS, inter-implantation site] are framed. (**I**) An insert shows the isolated portion of IIS myometrial section (as in H) between placentae, fixed, stained, and visualized as in (B), with all three channels superimposed. Myocytes from this section lack Kir7.1 expression. (**G**) The isolated portion of IS myometrial section (as in F) adjacent to placenta, fixed, stained, and visualized as in (B), with all three channels superimposed. Two regions are indicated: (**J**) that includes both circular and longitudinal myometrial tissue and shows Kir7.1 presence [(J) and (**K**), anti-Kir7.1, red], as well as apical endometrium/decidua surrounding the placenta (**L**) that also expresses Kir7.1 [(L) and (**M**), anti-Kir7.1, red].

The uterus is a hollow structure that, in rodents, comprises several strong layers of smooth muscle, including a longitudinal outer layer and a circular inner layer (Fig. 1A). The circular inner layer surrounds the endometrium, while the longitudinal layer aligns along the longitudinal axis of the uterus (*22*, *23*). As in all other smooth muscles, uterine contraction is initiated by the concerted action of various cation and voltage-gated ion channels that ultimately elevate calcium (Ca^2+^) in the myometrial cells activating actin-myosin complexes and initiating the cross-bridge cycle (*2*, *3*, *24*). Potassium (K^+^) channels, on the other hand, play a vital role in maintaining quiescence by hyperpolarizing the myolemma, thereby inactivating the voltage-dependent channels. Recently, a specific ion channel that plays an important role in uterine quiescence in both humans and mice has been identified: Kir7.1 (*17*, *25*–*27*). While global knockout of this gene is lethal (*27*), the knockdown of Kir7.1 increases uterine contractility, while reintroducing Kir7.1 after knockdown restores uterine quiescence (*17*). A decrease in myometrial Kir7.1 transcription coincides with the onset of labor (*17*). However, the endogenous regulation of Kir7.1 in the myometrium was not known. To study Kir7.1 functional expression and regulation during murine gestation, we combined detailed immunohistology with electrophysiological interrogation of myometrial cells isolated from murine uteri.

To activate Kir7.1 in myometrial myocytes, we used P4 that we previously identified as a potent and specific endogenous Kir7.1 activator in the choroid plexus and retinal pigment epithelia (*28*, *29*). Furthermore, to confirm specific activation of murine Kir7.1 by P4, we applied the murine Kir7.1 antagonist VU590 (*17*). First, we confirmed that Kir7.1 is not expressed in the murine myometrium of nonpregnant mice (Fig. 1, B to D). Functional absence of Kir7.1 was supported by both immunohistochemistry studies (Fig. 1, B to D) and by whole-cell electrophysiological recordings from freshly isolated myometrial cells (fig. S1, A to H). The isolated myocytes were subjected to a voltage ramp starting from a holding potential of 0 mV as shown in fig. S1, revealing residual potassium currents. Despite multiple repetitive attempts, neither Kir7.1-like conductance nor its P4-dependent current potentiation was observed.

We identified myometrial tissue by characteristic presence of α–smooth muscle actin (α-SMA; Fig. 1, B and C, and fig. S1, A, D, and E). Isolated myocytes from nonpregnant murine uteri lacked not only Kir7.1 protein expression (fig. S1C) but also ion channel activity (fig. S1, F to H). However, the presence of Kir7.1 was detected in murine myometrium isolated at 15.5 days post coitum (dpc), which corresponds to late gestation term (Fig. 1, E to G). Kir7.1 was not detected in myometrial tissue in the inter-implantation site (IIS), which is located between placentae (Fig. 1, E, H, and I). However, Kir7.1 expression was abundant in the uterine myocytes adjacent to the placenta, within the implantation site (IS; Fig. 1, E to K). As mentioned above, myometrial tissue was identified by the presence of α–smooth muscle actin (Fig. 1, I to L). As shown in Fig. 1 (G, J, and K), Kir7.1 was detected in both the longitudinal outer layer and the circular inner layer. Additionally, Kir7.1 was also present in decidua/endometrial tissue as shown in Fig. 1 (G, L, and M). Pregnancy-related myometrial Kir7.1 expression corroborates a previous report (*17*); however, its exclusive localization at the ISs of the uterus was unexpected.

Functional Kir7.1 expression in pregnant myometrium was also confirmed by immunocytochemistry and whole-cell electrophysiology from isolated uterine myocytes as shown in fig. S2 (A to F). These cells were positive for both α-SMA and Kir7.1 (fig. S2, A to C). There seems to be variable populations of Kir7.1-expressing myocytes with variable responses to P4 (fig. S2, D to F), perhaps due to a sensitivity of Kir7.1 to enzymatic digestion required to isolate individual myocytes. Whole-cell patch-clamp recordings from isolated myocytes revealed a conductance that resembled Kir7.1 inwardly rectifying potassium current, which was further potentiated by 10 μM P4 and inhibited by the Kir7.1-antagonist VU590 (fig. S2, D to F). To exclude potential involvement of ERG (Ether-a-go-go-Related Gene, also known as *KCNH2*, which codes for Kv11.1) channels, P4-stimulated myocytes were exposed to 1 μM dofetilide (DOF), a potent ERG channel inhibitor (fig. S2D). As demonstrated in fig. S2 (D to F), DOF did not alter P4 potentiation of Kir7.1, indicating minimal, if any, involvement of ERG activity in the P4 effect on Kir7.1.

**Fig. 2.**
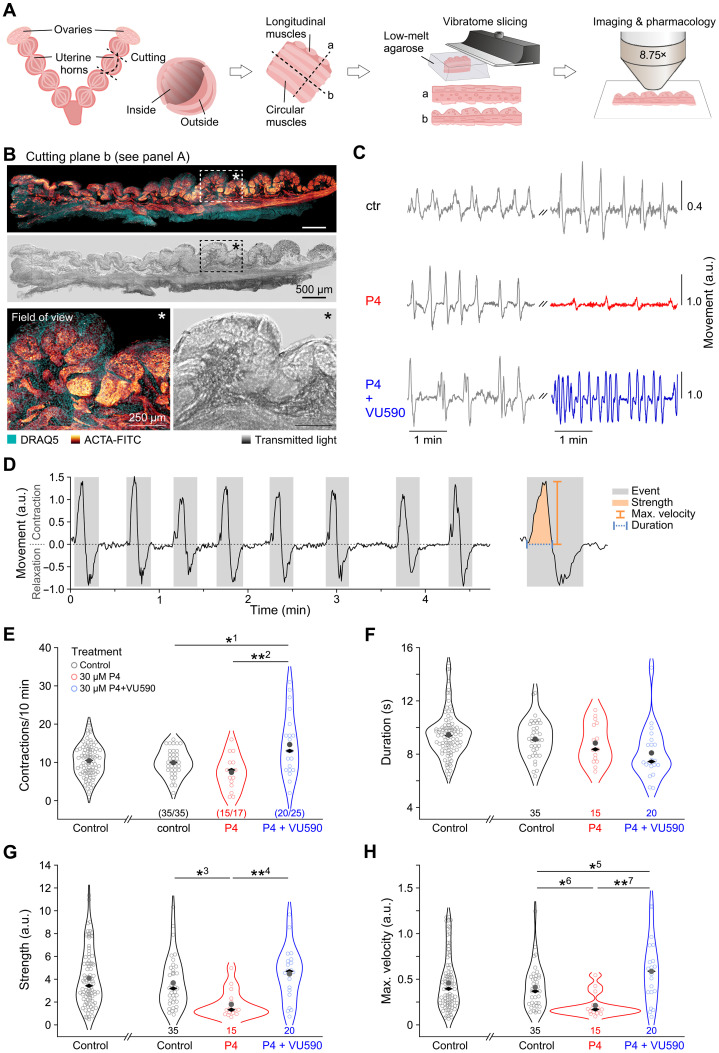
Progesterone and VU590 antagonistically affect myometrial contractions. (**A**) Schematic illustrating the experimental approach. Blocks of dissected murine myometrium (14.5 dpc) were embedded in agarose and slices were cut along the axes of either the longitudinal (a) or circular (b) muscles. (**B**) The latter were immunostained against α-SMA (ACTA-FITC). Top color micrograph: Confocal fluorescence indicating smooth muscle. Nuclei are stained with DRAQ5 (cyan). Longitudinal muscles are cut transversally. Bottom micrograph: Corresponding transmitted light image. Rectangles with asterisks mark the area of higher magnification below that represents field of view in time-lapse recordings. (**C**) Representative traces (pixel displacement versus time) depicting spontaneous myometrial contractions under control condition (left) and during a later period under three different conditions (right): (i) control (ctr; top; gray); (ii) 30 μM P4 incubation (middle; red); and (iii) simultaneous incubation with 30 μM P4 and 20 μM VU590 (bottom; blue). (**D**) Representative time-lapse recording of myometrial contractility [pixel displacement in arbitrary units (a.u.) versus time]. Dotted line indicates resting state. Individual contraction-relaxation events (gray boxes) are characterized by successive peaks in opposite direction. Right: A single event to illustrate analysis parameters, i.e., contraction strength (orange area), maximum velocity (orange line), and duration (blue dotted line) of contractions. (**E** to **H**) Quantification of myometrial contractions in the absence or presence of either 30 μM P4 or 30 μM P4 and 20 to 100 μM VU590. Violin plots display data from individual regions of interest (ROIs; circles; *n* denoted below violins) as well as means (gray filled circles) and medians (black diamonds). (E) Contraction count per 10-min recording. (F) Duration of myometrial contractions. (C to E) Each circle represents the mean of several events. (G) Contraction strength as calculated from the area under curve in a.u. (H) Maximal contraction velocity in a.u. Asterisks denote statistical significance (*^1^*P* = 0.008; **^2^*P* = 0.001; *^3^*P* = 0.015; **^4^*P* = 0.001; *^5^*P* = 0.05; *^6^*P* = 0.041; **^7^*P* = 0.0002).

To further explore P4 activation of Kir7.1 in uteri of pregnant mice, we performed in situ measurement of uterine myometrial contractions in acute tissue slices as described in Fig. 2 (A to C). The myometrial contractility was monitored under three different conditions: (i) control condition, to measure spontaneous myometrial contractions; (ii) treatment condition #1, i.e., in presence of P4 (30 μM); and (iii) treatment condition #2, i.e., during simultaneous incubation with P4 (30 μM) and VU590 (20 to 100 μM) (Fig. 2 and movies S1 to S3). As expected from electrophysiological profiling (fig. S2), P4 and VU590 antagonistically affected various myometrial contraction parameters (Fig. 2D). First, exposure to VU590 (condition #2) statistically increased the number of contractions compared to both control and P4-treatment conditions (Fig. 2E). The duration of contractions was not changed, which is expected because this parameter is also controlled by calcium transporters, as well as by other ion channels, including voltage-gated K^+^ channels (Fig. 2F). Notably, P4 reduced contraction strength and velocity, while exposure to VU590 in presence of P4 completely reversed these effects (Fig. 2, G and H).

These results revealed that P4 maintains myometrial quiescence via its effect on Kir7.1. Given the fact that the mammalian placenta produces P4 during pregnancy, the specific expression of Kir7.1 at the placental site of the uterus and its activation by P4 is physiologically relevant. While the aforementioned properties of Kir7.1 were demonstrated using a mouse model, we next decided to explore whether human Kir7.1 is similarly regulated, especially because the human placenta is the main P4 producer in the body during pregnancy (*7*, *30*).

### Progesterone activates human and murine Kir7.1 in a similar manner

**Fig. 3.**
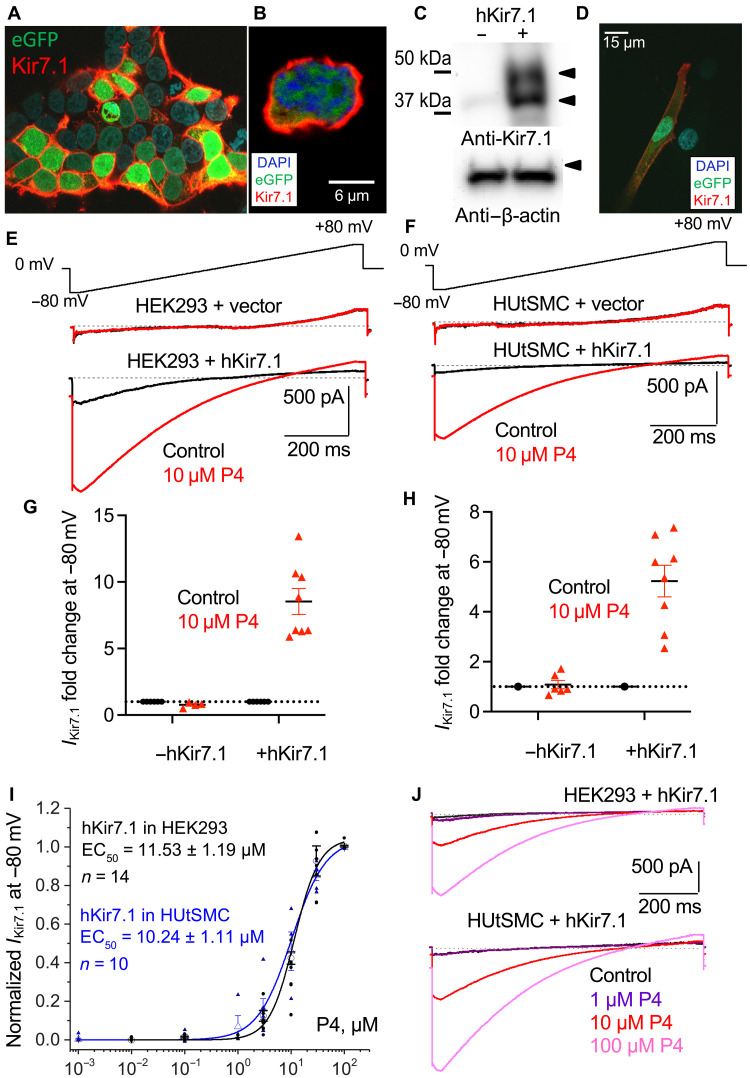
Progesterone activates recombinant hKir7.1 expressed in myometrial cells as well as in HEK293 cells. (**A**) Immunocytochemistry of recombinant hKir7.1 (red) in HEK293 cells visualized with anti-Kir7.1 antibody. (**B**) Individual HEK293 cell transfected as described in (A) shows typical membrane localization of hKir7.1 (red). (**C**) Western blot of the protein lysates isolated from HEK293 cells transfected with either: (i) empty vector [pIRES2-eGFP; (−); no Kir7.1 signal] or (ii) pIRES2-eGFP-hKir7.1 [+; glycosylated, and, likely, a shorter immature isoform of Kir7.1 is seen]. The blot was probed with anti-Kir7.1 antibody, and β-actin was used as loading control. (**D**) Immunocytochemistry of human uterine smooth muscle cells (HUtSMCs) transfected with pIRES2-eGFP-hKir7.1. (**E**) Representative traces of hKir7.1-derived potassium conductance recorded from HEK293 cells transfected with either empty vector (top) or hKir7.1 (bottom). (**F**) Representative traces of hKir7.1-derived potassium conductance recorded from HUtSMCs transfected with either empty vector (top) or hKir7.1 (bottom). (**G** and **H**) The average current fold increase (FI) recorded at −80 mV for recombinant hKir7.1 expressed either in HEK293 (G) or HUtSMCs (H). Transfection with a pIRES2-eGFP-hKir7.1 showed 5- to 10-fold potentiation of the currents after cells were stimulated with 10 μM P4. The cells transfected with the empty vector do not respond to P4 or display hKir7.1 current. (**I** and **J**) Dose-response relationships and corresponding EC_50_ values (I) for P4 activation recorded from two different cell lines, HEK293 or HUtSMCs, as well as representative recordings (J) transiently transfected with pIRES2-eGFP-hKir7.1. Potassium currents depicted on (E) to (J) were recorded in response to an indicated voltage ramp. Dose-response curves were calculated using the average current density at −80 mV for *n* = 14 and *n* = 10 cells for HEK293 and HUtSMCs, respectively. EC_50_ for P4 obtained from both conditions were similar. Data are means ± SEM.

Isolation of murine uterine myocytes for electrophysiological interrogation requires mild proteolytic digestion of the uteroplacental unit. This procedure may lead to a partial incapacitation of Kir7.1 due to a prolonged exposure to proteolytic enzymes and, hence, result in notable variabilities among recorded currents as shown in fig. S2 (D to F). It is also possible that Kir7.1 expression in gestational myocytes varies between cells. To reduce Kir7.1 response variability, a heterologous expression system was used to overexpress both murine and human Kir7.1 in human embryonic kidney (HEK) 293 cells (Fig. 3, A to C, and fig. S3). Additionally, homologous expression system for transient expression of hKir7.1 in primary human uterine smooth muscle cells (HUtSMCs) was used (Fig. 3D).

Both recombinant murine and human Kir7.1 were potentiated by P4 (fig. S3, A to C). Kir7.1 is an evolutionarily conserved ion channel with 92.6% sequence identity between humans and rodents (mouse and rat). Given this homology, it is expected for Kir7.1 to exert similar biological functions in both murine and human tissues. Human Kir7.1 expressed in either HEK293 or cultured human myometrial cells was potentiated by P4, while no such effect was observed when P4 was applied to cells transfected with empty vector (Fig. 3, E to H, and fig. S3, A and B).

The median effective concentration (EC_50_) of P4 potentiation of Kir7.1 was identical between both murine Kir7.1 expressed in HEK293 cells (fig. S3C) and human Kir7.1 expressed in either HEK293 or HUtSMCs, with values ranging between 9.4 and 11.5 μM (Fig. 3, I and J, and fig. S3C). This is in line with physiological local P4 concentrations that are reached during pregnancy (*7*), where P4 is synthesized in both the uterus and placenta. The human placenta produces 250 mg per day of P4 (*7*), which leads to substantially greater local steroid concentrations than blood plasma concentrations (*30*). Therefore, endogenous P4 not only is locally produced in the myometrium and at the IS but also is available at physiological concentrations to activate Kir7.1.

### Human Kir7.1 responds to select endogenous steroids

**Fig. 4.**
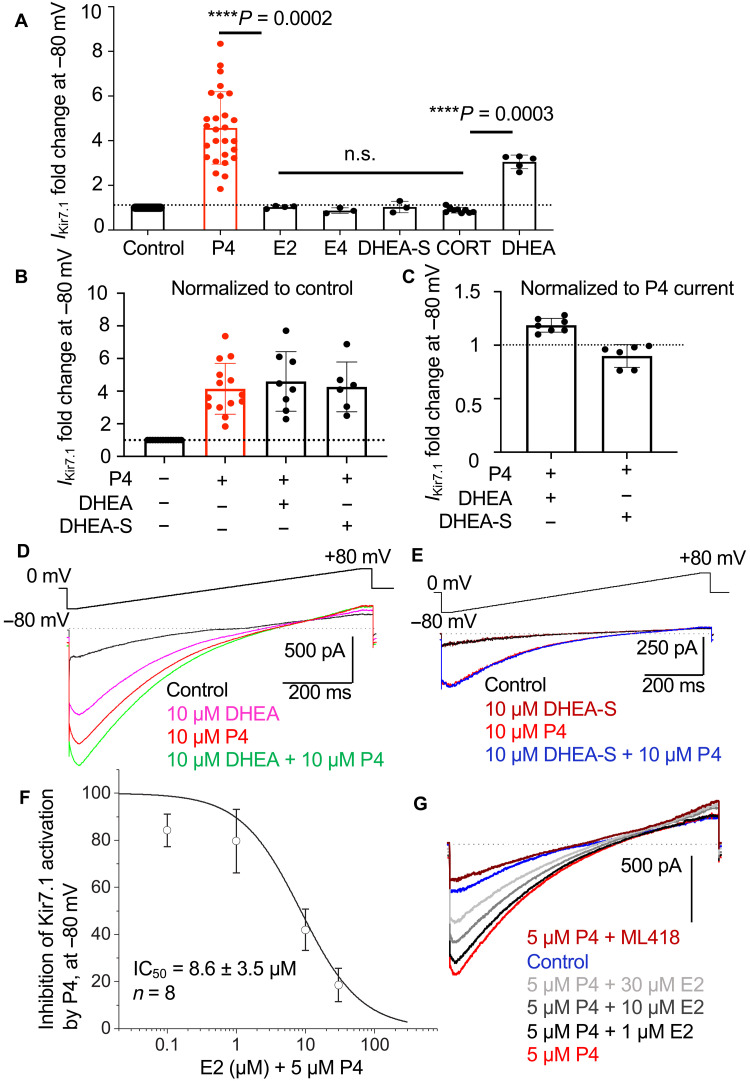
Potentiation of hKir7.1 by endogenous steroids. (**A**) The average current FI of hKir7.1 at −80 mV recorded from recombinant protein expressed in HEK293 cells that were exposed to 10 μM of the following compounds: P4, E2, estetrol (E4), DHEA-S, cortisol (CORT), and DHEA. Statistics are as follows: control: *n* = 29; P4: 4.57 ± 0.31, *n* = 27; E2: 1.03 ± 0.03, *n* = 4; E4: 0.87 ± 0.07, *n* = 3; DHEA-S: 1.03 ± 0.15, *n* = 3; cortisol: 0.89 ± 0.04, *n* = 9; and DHEA: 3.05 ± 0.14, *n* = 5. Amplitudes were normalized to control. Asterisks denote statistical significance and the corresponding *P* values calculated using nonparametric Kruskal-Wallis test; n.s. stands for nonsignificant. (**B**) The average current FI of recombinant hKir7.1 at −80 mV recorded from HEK293 cells that were exposed to the combination of steroids indicated. There were no differences regarding the order in which steroids were applied first. However, the combination of P4 with either DHEA or DHEA-S was always applied last. P4 and DHEA were able to activate hKir7.1, while DHEA-S did not change hKir7.1 response. Statistics are as follows: control: *n* = 14; P4: 4.14 ± 0.42, *n* = 14; P4 + DHEA: 4.60 ± 0.65, *n* = 8; and P4 + DHEA-S: 4.27 ± 0.62, *n* = 6. Amplitudes were normalized to control. (**C**) Current densities of combined P4/DHEA or P4/DHEA-S were normalized to P4-responses. Statistics are as follows: P4 + DHEA: 1.19 ± 0.02, *n* = 7; and P4 + DHEA-S: 0.86 ± 0.06, *n* = 4. (**D** and **E**) Representative traces of hKir7.1-derived currents for experiments depicted in (B) and (C). (**F**) The inhibition of P4-activation of hKir7.1 by E2. Median inhibitory concentration (IC_50_) of E2 inhibition was calculated using the average current density obtained at −80 mV for eight cells recorded from hKir7.1-expressing HEK293. (**G**) Representative traces for (F). To confirm that P4 and steroids activate hKir7.1-derived potassium conductance, a specific inhibitor for hKir7.1, ML418 was used. Data are means ± SEM.

While few specific synthetic inhibitors of human Kir7.1 are known, such as ML418 (*31*), both endogenous activators (except for P4), as well as synthetic activators, had yet to be identified. Therefore, the modulation of human Kir7.1 by additional pregnancy-related steroids was explored. While P4 plays a dominant role in maintaining quiescence during pregnancy to accommodate the developing fetus, there are other pregnancy-related hormones that play unique, yet poorly understood, roles. DHEA and its sulfated form, DHEA sulfate (DHEA-S), are produced by fetal and maternal adrenals in large amounts for placental E2 production (*32*). In the placenta, E2 is the most abundant estrogen synthesized. According to our previously reported data, murine Kir7.1-activation is P4 specific (*28*, *29*). However, steroid specificity of human Kir7.1 (hKir7.1) was not known. To evaluate its specificity, recombinant hKir7.1 was stimulated with various endogenous steroids (Fig. 4 and fig. S4). There was no significant increase in potassium conductance of hKir7.1 recorded from HEK293 cells that were exposed to 10 μM E2, estetrol (E4), DHEA-S, and cortisol (Fig. 4A and fig. S4), while exposure to P4 caused a 4.5-fold increase in hKir7.1 activity (Fig. 4A). Another steroid, DHEA, also displayed agonistic activity by increasing hKir7.1 activity ~3-fold (Fig. 4A and fig. S4), while its membrane-impermeable sulfated analog, DHEA-S, was inactive (Fig. 4, A and B, and fig. S4). This indicates that hKir7.1 might be activated by membrane-permeable steroids from the intracellular side. Additionally, DHEA and P4 applied together showed a cumulative mode of action (Fig. 4, C to E). While E2 did not activate hKir7.1 upon direct administration (Fig. 4A and fig. S4), it was able to antagonize the P4 effect when added in combination (Fig. 4, F and G). The median inhibitory concentration (IC_50_) of the E2 inhibition was 8.6 μM, indicating a putative antagonistic mode of action between E2 and P4, corroborating previous studies showing the inverse effects of P4 and E2 on uterine contractions (*5*, *10*). To confirm that P4-activated currents were carried by hKir7.1channel, we applied ML418, a specific irreversible Kir7.1 antagonist (*31*), at the end of recordings to block both P4-activated and basal Kir7.1 currents (Fig. 4G).

While P4 has been recognized as a potent inhibitor of uterine contractions when administered in micromolar concentrations, E2 transforms the myometrium into an active, contractile state (*10*–*13*, *18*, *33*). The effects of both P4 and E2 on myometrial tissue have been widely recognized as a rapid, nongenomic phenomenon, indicating that classical genomic steroid receptors that act via changes in gene expression are not the only regulatory units in these cases (*11*, *18*, *34*). Our data show that hKir7.1, an ion channel previously shown to be vital for myometrial quiescence (*17*), is activated by P4, and this effect is antagonized by E2, revealing an unexpected mechanism for P4 functional withdrawal in primates.

### hKir7.1 is activated by synthetic steroids used to treat preterm labor

**Fig. 5.**
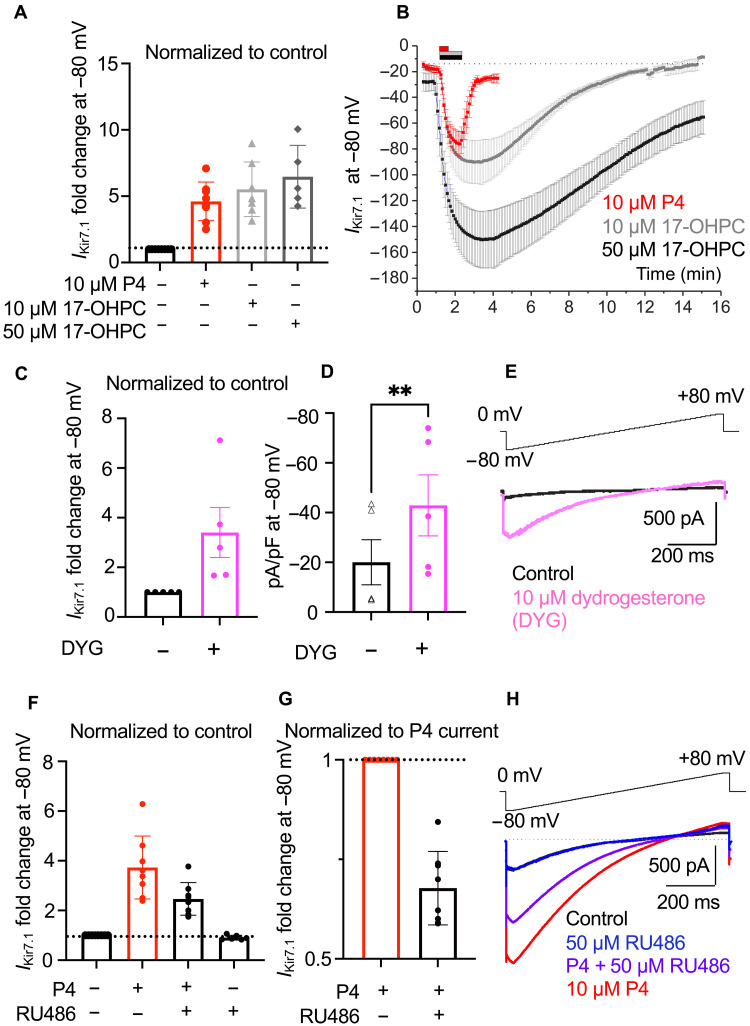
Regulation of hKir7.1 by synthetic steroids. (**A**) The average current FI of recombinant hKir7.1 at −80 mV recorded from HEK293 cells exposed to either 10 μM P4 or 10 μM and 50 μM 17-OHPC. Control: *n* = 8; P4: 4.61 ± 0.51, *n* = 8; 10 μM 17-OHPC: 5.52 ± 0.78, *n* = 7; and 50 μM 17-OHPC: 6.47 ± 1.06, *n* = 5. (**B**) Time course of steroid activation and washout. Averaged Kir7.1 current amplitudes from HEK293 cells as described in (A) obtained at −80 mV were plotted against time to show a fast response to P4 (red) and its fast washout. The responses to 10 μM 17-OHPC were slower and required a much longer washout. The time of compound’s application to the bath solution is indicated by the bars above. (**C**) The average current FI of hKir7.1 at −80 mV after exposure to 10 μM dydrogesterone (DYG). Control: *n* = 5; and DYG: 3.40 ± 1.01, *n* = 5. Amplitudes were normalized to control. (**D**) Current densities as described in (C); control: −20.05 ± 9.07, *n* = 5; and DYG: −42.89 ± 12.25, *n* = 5. (**E**) Representative hKir7.1 traces recorded as mentioned in (C). (**F**) The average current FI of hKir7.1 obtained at −80 mV from transfected HEK293 after exposure to 10 μM P4 alone, in the presence of 50 μM RU486, or the combination of both. Control: *n* = 8; P4: 3.73 ± 0.45, *n* = 8; P4 + RU486: 2.46 ± 0.23, *n* = 8; and RU486: 0.91 ± 0.04, *n* = 6. Amplitudes are normalized to control. (**G**) The averaged amplitude of cellular response to P4 and RU486 combination from (F) was normalized to the amplitude of P4 response. Control: *n* = 8; and P4 + RU486: 0.68 ± 0.03, *n* = 8. (**H**) The representative recombinant hKir7.1 responses from HEK293 cells after exposure to 50 μM RU486 with or without P4. **P ≤ 0.01 calculated with paired *t* test. Data are means ± SEM.

Until recently, vaginal administration of P4 or intramuscular injections of synthetic progestin, 17-OHPC (Makena), were the only approved treatments for the prevention of preterm labor (*35*, *36*). Both treatments were expected to act via activation of the nuclear P4 receptor. To explore whether 17-OHPC has any impact on hKir7.1, we compared its effects on hKir7.1 expressed in HEK293 cells with the P4 effect (Fig. 5, A and B). 17-OHPC not only specifically and potently activated hKir7.1 but also had a much stronger membrane retention, as demonstrated by a notably longer washout time (Fig. 5B and fig. S5, A and B). Our results show that hKir7.1 potentiation by 10 μM 17-OHPC was similar in strength to that of 10 μM P4; however, drug washout took five times longer for 10 μM 17-OHPC and, after exposure to a higher concentration (50 μM), 17-OHPC was never fully washed out (Fig. 5, A and B, and fig. S5, A and B). This could explain why 17-OHPC has been shown to be effective with weekly injections, while vaginal P4 needs to be administered daily (*37*).

Another promising treatment for preterm labor prevention now in clinical trials is the synthetic progesterone, dydrogesterone (fig. S5D). Dydrogesterone has been used worldwide since the 1960s in the treatment of threatened and recurrent miscarriages. Moreover, the drug was recently found to inhibit uterine contractions in a nongenomic manner (*38*–*40*). Here, we showed that hKir7.1 potentiation by dydrogesterone is similar to that of P4 (Fig. 5, C to E), further confirming the role of hKir7.1 in the fast, nongenomic regulation of uterine contractions. Dydrogesterone is also used to treat menstrual cramps, in which case the drug may also exert its effects via myometrial ion channels.

### Kir7.1 potentiation by progesterone is attenuated by the abortifacient drug mifepristone

Exogenous administration of 200 to 600 mg of the antiprogestin mifepristone (RU486) alone induces contractions, thereby mimicking the onset of labor (*41*). RU486 (fig. S5E) has also been shown to increase uterine sensitivity to prostaglandins and, when taken in succession, increases the efficacy of early pregnancy termination from 60 to 95% (*18*), making it a well-known abortifacient with an unclear mode of action. The application of RU486 to human Kir7.1 expressed in HEK293 cells revealed its antagonistic mode of action (Fig. 5, F to H, and fig. S5F). When introduced alone to human Kir7.1-expressing HEK293 cells, at high micromolar concentrations, RU486 did not alter Kir7.1 conductance (Fig. 5, F and H). However, RU486 was able to interfere with the P4 potentiation of Kir7.1 when given concurrently, resulting in the inhibition of P4-driven Kir7.1 activation (Fig. 5, F to H, and fig. S5F) and, therefore, uncovering the previously unknown mode of action for this drug.

### Kir7.1 is functionally expressed in placental pericytes

**Fig. 6.**
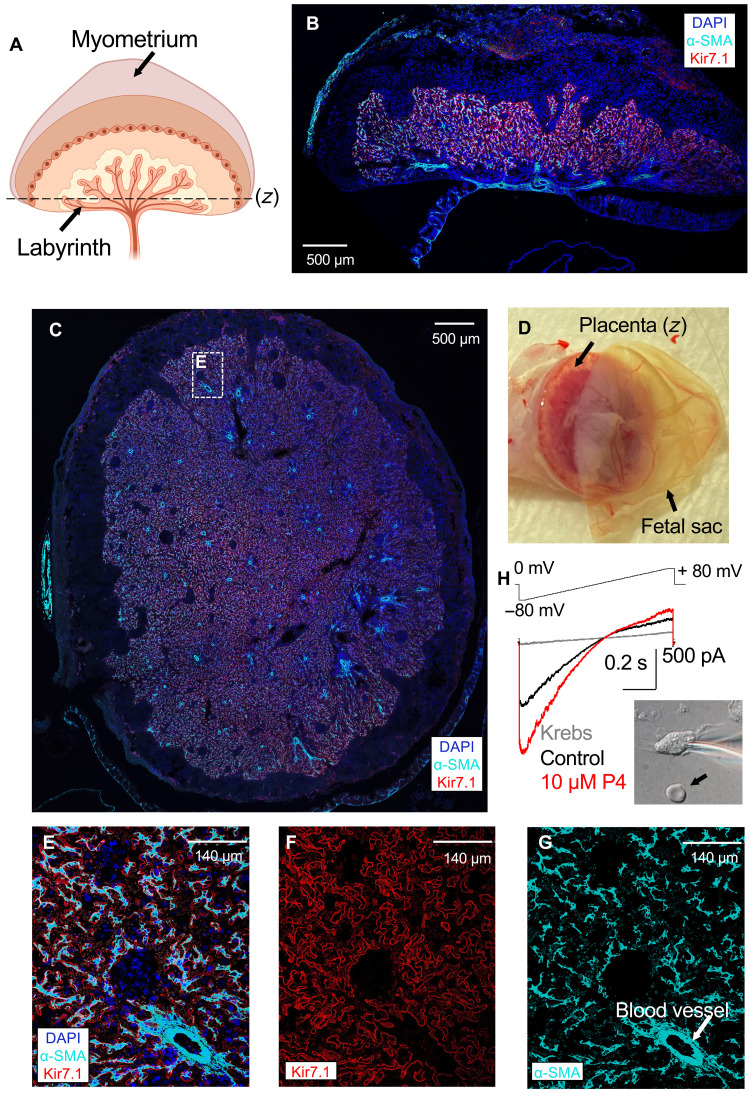
Kir7.1 expression and localization in the murine placental labyrinth. (**A**) Schematic of the uteroplacental unit. The labyrinth and the horizontal section plane (*z*) are shown. Created with BioRender.com. (**B**) Immunostaining of the coronal section of murine uteroplacental unit isolated from 15.5 dpc. The coronal section was co-stained with anti–α-SMA antibody (cyan), anti-Kir7.1 antibody (red), and DAPI (blue, indicating nuclei). An intense Kir.7.1 signal (red) was detected in the placental labyrinth. (**C**) Immunostaining of the horizontal cross section through labyrinth through the plane (z) as shown in (A) collected at 15.5 dpc. The section was co-stained as described in (B). Kir7.1 (red) signal was co-localized with intense signal from non-vascular α-SMA containing cells (cyan). (**D**) Isolated murine uteroplacental unit with placenta and fetal sac indicated. Plane (*z*) is indicated as in (A). (**E**) Zoomed-in portion placental labyrinth as indicated in dotted frame in (C). Placental labyrinth co-stained as in (B) with all three channels superimposed. Red and cyan signals confirm that Kir7.1 is expressed in pericytes but absent from vascular smooth muscles. Cell nuclei are stained by DAPI (blue). (**F**) Inset E from (C) shows strong Kir7.1 staining (red) with typical membrane localization of the ion channel. (**G**) Same section as in (E) visualized with anti–α-SMA (cyan) antibodies reveals a number of cells with pleomorphic morphology typical for placental pericytes. Additionally, vascular smooth muscles surrounding blood vessels (cyan, indicated by arrow) are also shown. (**H**) Representative Kir7.1 current recorded from isolated placental pericytes (differential interference contrast image of the cell is shown in the insert, and arrow indicates red blood cell). This recording shows characteristic inward rectification typical for Kir7.1 that is further stimulated by exposure to 10 μM P4.

Serendipitously, we found an additional organ with high Kir7.1 expression during late gestation: the placenta. Placentae, isolated from five 15.5-dpc mice, were examined for Kir7.1 expression, which was particularly intense in the murine placental labyrinth (Fig. 6, A to D), the main interface between maternal and fetal blood exchange in mice. The labyrinth is a structure that assembles during late gestation and contains an elaborate network of villi that maintain gas and nutrient transport from maternal to fetal circulation, as well as waste removal (*8*, *42*). One of the least studied cellular components of the labyrinth are placental pericytes, specific mural cells that control the blood supply to the fetus through a capillary network (Fig. 6, E to G) (*8*). Placental pericytes can be identified by characteristic presence of α-SMA (*8*, *43*) (Fig. 6, B, C, E, and G), as well as by neural/glial antigen 2 (NG2; fig. S9), a marker that is expressed in nascent microvessels surrounded by vascular pericytes.

Kir7.1 expression was identified in placental pericytes in labyrinthic sections from 15.5-dpc placenta (Fig. 6, C and E to G, and figs. S6 and S9, A, B, and E). At the same time, vascular myocytes, which surround placental arterioles and veins and are also visualized by α-SMA, and NG2 lacked Kir7.1 expression (Fig. 6, E to G, and figs. S6 and S9, A, C, and E). Specifically, Kir7.1 was detected in the membrane junctions between pericytes and vascular myocytes (fig. S6C), resembling intimate pericytial-muscular contacts in the brain microvasculature (*44*, *45*). The functional presence of Kir7.1 in pericytes was further confirmed by direct electrophysiological recordings from isolated murine placental pericytes (Fig. 6H and fig. S7). As shown in fig. S7, not only pericytes expressed functional Kir7.1, which was activated by P4, but also this activation was removed via exposure to the selective murine Kir7.1 inhibitor, VU590. The isolated pericytes revealed asymmetrically clustered Kir7.1 expression (fig. S6, A and B). Additionally, Kir7.1 presence at the protein level was detected in human placenta (fig. S9F).

Dysfunction in placental pericytes is emerging as an important factor linked to preeclampsia, a syndrome with clinical manifestations such as severe hypertension and edema, and increased risk factor for preterm labor (*19*, *20*, *46*). Placental pericytes are contractile mural cells that control the blood supply to the fetus and tightly enwrap placental capillaries, visualized by their marker protein CD31 (fig. S8) and NG2 (fig. S9). As mentioned above, placental pericytes also form connections to the vasculature of the arteries and veins (Fig. 6E and fig. S8), and, hence, pericytes can share their membrane potential with the membrane potential of the vascular smooth muscle. The network of pericytes connected to the vascular smooth muscles can, therefore, efficiently control vasodilation via propagation of hyperpolarizing stimuli. K^+^ channels, such as Kir7.1, play a vital role in maintaining cellular hyperpolarization. Like its function in uterine contractility (*2*, *17*), pericytial Kir7.1 could, therefore, control membrane potential of the pericytial network and serve as a regulatory element of placental vasodilation. Thus, pericytial Kir7.1 up-regulation by P4 is likely to keep the cells in a relaxed, hyperpolarized state and ensure transmission of this state to the vascular smooth muscles, hence controlling the blood exchange between maternal and fetal sides and promoting more effective fluid exchange.

Given that placental pericytes dysfunction is linked to preeclampsia (*8*) and the fact that dydrogesterone and 17-OHPC are now being investigated as a potential therapeutic intervention for preeclampsia (*19*, *20*), our results support the notion that Kir7.1 is a promising molecular target to potently control uteroplacental physiology.

## DISCUSSION

Preterm birth is defined as birth less than 37 weeks of gestation and accounts for 85% of perinatal morbidity and mortality (*47*, *48*). Annually, the societal cost of preterm births is $25.2 billion (*48*). Preterm labor poses a substantial threat to maternal well-being and the health of the baby. Now, preterm birth rates are on the rise in the United States with ~10% of all pregnancies ending in early deliveries (*47*). Sadly, this translates to almost 450,000 babies born prematurely every year in the United States, which, on a global scale, amount to 15 million babies. Preterm birth has a disproportionate impact on communities of color and especially affects African American women, whose preterm birth (14.6%) rate is 50% higher than those of other racial cohorts (*47*). Until recently, vaginal administration of P4 or intramuscular injections of the synthetic progestin 17-OHPC (Makena) were the only approved treatments for the prevention of preterm labor (*35*, *36*). Intramuscular administration of 250 mg of 17-OHPC on a weekly basis starting at 16 to 20 weeks until delivery has been shown to substantially reduce the rate of preterm delivery (*36*). Recently, however, larger studies have shown a less pronounced reduction in preterm labor (*49*). This led the Food and Drug Administration (FDA) to withdraw Makena in 2023, despite benefiting a certain proportion of pregnant patients (*50*, *51*). As of now, except for P4, there is no FDA-approved pharmaceutical intervention to reduce the risk of premature birth. Clearly, we still lack a basic understanding of the biological processes underlying preterm births. Therefore, a deeper conceptual understanding of the mechanisms controlling uterine quiescence, as well as contractions, is essential for the prevention of preterm labor.

Kir7.1 was previously identified as an important ion channel in maintaining membrane hyperpolarization and uterine quiescence during late gestation in both rodent and human myometrium (*17*). However, the endogenous regulation of this channel was unknown. In this study, we not only determined Kir7.1 expression, localization, and function during pregnancy but also revealed the physiological function of Kir7.1 in uterine quiescence through its regulation by exogenous and endogenous steroids known to induce or inhibit uterine contractions.

Myometrial Kir7.1 expression displayed a surprising localization at the ISs of the uterus, i.e., in a proximity to placenta, the organ with active steroidogenesis and, hence, progesterone production. It is possible, therefore, that Kir7.1 expression is also regulated by placental steroids, in addition to their direct effect on Kir7.1 channel activity.

While P4 has been well recognized as one of the main steroid hormones essential for pregnancy maintenance and gestational myometrial quiescence, its role in the onset of labor was not well understood because of a phenomenon known as functional withdrawal (*5*, *6*, *9*, *14*, *15*, *52*). While, in rodents, the onset of labor coincides with sharp decline in P4 levels, the parturition in primates begins while the blood plasma retains its high P4 (*5*, *14*). Therefore, the inability of primate myometrium to maintain quiescence during parturition would require either removal or inhibition of P4 targets that suppress myometrial contractility. While P4 nuclear receptor should ultimately play an important role, its level stays either unchanged or elevated during pregnancy and during parturition (*14*). This finding led to a hypothesis that another target, such as nongenomic P4 receptor, i.e., an ion channel, could be involved. The onset of labor coincides with a decrease in Kir7.1 (*17*). Moreover, as we have shown above, P4 effect on human Kir7.1 can be antagonized by an exposure to micromolar levels of E2. The levels of E2 are rising before parturition in most mammals (*14*, *52*), increasing up to 30-fold by birth (*53*). The local E2 concentration in myometrium, especially in the region adjacent to placenta, could be even higher, which would expose Kir7.1 to E2 concentrations able to antagonize its activation by P4, i.e., IC_50_ of 8.6 μM. The competitive effect of E2 toward Kir7.1 would result in its functional inactivation and inability to sustain myometrial quiescence. This mechanism can contribute to a functional withdrawal, an unexplained phenomenon in human obstetrics.

Additionally, we revealed an unexpected mechanism behind the effect of the abortifacient drug, mifepristone (RU486), which is the first and only FDA-approved drug for early pregnancy termination. RU486 has been administered to over 5.9 million Americans in the past two decades (*41*, *54*); however, the exact mechanisms of action have not been elucidated. The administration of 50 μM RU486 to human Kir7.1 led to 40% reduction in Kir7.1 activation by P4, uncovering mifepristone’s antagonistic and nongenomic mode of action at Kir7.1. These data show that RU486 affects Kir7.1 and, consequently, interferes with P4-mediated myometrial relaxation. An exposure to RU486 can, thus, lead to uterine contractility and pregnancy termination via its effect on Kir7.1. While the antagonistic effect of RU486 toward nuclear P4 receptor has been extensively studied and is well-known (*21*), our results reveal an additional fast nongenomic mode of action of this drug.

An additional risk factor for preterm birth is preeclampsia, a syndrome with clinical manifestations such as severe hypertension and edema. If left untreated, then preeclampsia leads to fetal and maternal mortality. This serious medical condition can arise from placental insufficiency and impaired uteroplacental fluid perfusion (*8*), which, in the United States, affects between 5 and 8% of pregnancies (*46*). According to literature (*55*), the rates of gestational hypertension/mild preeclampsia among non-Hispanic black women increased from 1.8 to 4.7%, while severe preeclampsia has risen from 0.7 to 3.6% from 1996 to 2014 among the same population cohort. P4 and progestogens have been explored as a treatment against preeclampsia since the 20th century, and recent studies indicated that dydrogesterone treatment showed statistically significant benefits (*19*, *20*, *38*–*40*). One of the emerging factors contributing to preeclampsia is dysfunction of placental pericytes (*8*). Placental pericytes are contractile mural cells that control the blood supply to the fetus and tightly enwrap microvessels to form connections to the vasculature of the arteries and veins. Hence, they are ideally positioned to control the blood exchange between maternal and fetal sites. The absence of P4 or pericytial insufficiency could alter this flow exchange by redistributing a large volume of placental blood back into maternal circulation, causing hypertension and edema while also producing fetal hypoxia. According to our data, murine placental pericytes functionally express P4-activated Kir7.1 that was detected in the membrane junctions between pericytes and vascular myocytes. According to the Human Protein Atlas, Kir7.1 is also expressed in human placenta (*56*). Kir7.1 presence at the protein levels was detected in human placenta (fig. S9F). Further studies are needed to test our hypothesis that pericytial Kir7.1 modulates placental vasculature and blood distribution and to evaluate the functional link between P4 and Kir7.1 in relation to pericyte insufficiency during preeclampsia.

Together, this study points to an important role of Kir7.1 in the nongenomic regulation of both endogenous and exogenous steroid effects during pregnancy, particularly during middle to late gestation. Our findings, therefore, deepen the understanding of uterine contractility and pave the way to develop selective pharmacological interventions to treat preterm labor by targeting potassium ion channels.

## MATERIALS AND METHODS

### Experimental design

#### Animals

The C57BL/6N (Charles River Laboratories) mice were kept at the animal facility of the University of California, Berkeley, in a room with controlled light (14 hours of light, 10 hours of darkness) and temperature (23° ± 0.5°C). The mice were fed a standard chow diet (PicoLab Rodent diet 20, LabDiet, 5053) and hyper-chlorinated water ad libitum. Animals were euthanized by CO_2_ and cervical dislocation according to the approved protocols. All experiments were performed in accordance with National Institutes of Health’s Guidelines for Animal Research and approved by University of California, Berkeley, and Washington University in St. Louis, School of Medicine Animal Care and Use Committee (AUP 2015-07-7742-2 and 22-0251), with every effort made to minimize suffering for the animals. All experiments used C57BL/6 N unless otherwise noted.

C57BL/6J mice used in experiments described in Fig. 2 and movies S1 to S3 were obtained from Charles River Laboratories, Sulzfeld, Germany; RRID: IMSR_JAX:000664. All animal procedures were approved by local authorities and in compliance with both European Union legislation (Directive 2010/63/EU) and recommendations by the Federation of European Laboratory Animal Science Associations. Mice were housed in littermate groups [room temperature (RT); 12-hour:12-hour light-dark cycle; food and water available ad libitum]. If not stated otherwise, tissue from pregnant mice at day 14.5 of gestation was used. Mice were killed by intraperitoneal injection of ketamine (200 mg/kg) and xylazine (25 mg/kg) and decapitation using sharp surgical scissors.

#### Myometrial smooth muscle isolation and primary cell culture

The uteri were dissected into 37°C Dulbecco’s phosphate-buffered saline [DPBS; without Ca^2+^ and Mg^2+^ (Thermo Fisher Scientific, 14190144)]. The tissue was then cut in ~1-mm^3^ pieces and digested in Pronase (0.1 mg/ml in DPBS; Sigma-Aldrich, 10165921001) at 37°C for 30 min in 5% CO^2^ atmosphere. Tissue was vigorously pipetted up and down every 10 min to help with the removal of endometrial cells. The reaction was inhibited by adding a 5× volume of fresh growth medium [high-glucose Dulbecco’s modified Eagle’s medium (DMEM) with l-glutamine (Thermo Fisher Scientific, 11965092) supplemented with 10% fetal bovine serum (FBS; X&Y Cell Culture, FBS-500-HI) and 1% PenStrep (Thermo Fisher Scientific, 15140-122)]. The remaining tissue was allowed to sediment for 1 min after which the supernatant was removed. The remaining tissue was further dissociated by trituration (50 times) in 37°C TrypLE Express (Thermo Fisher Scientific, 12605010). After the cell clusters had sedimented, the supernatant was transferred to a 5× volume of fresh growth medium. The uterine cells were collected by centrifugation at 300*g* for 5 min, resuspended in fresh growth medium, and plated on 5-mm glass coverslip in a four-well plate. The cells were allowed to attach to the coverslip for 2 hours at 37°C and 5% CO_2_ before electrophysiology measurements.

#### Human postpartum placenta

Human postpartum placenta biospecimen (1 g of tissue) was commercially obtained from Discovery Life Sciences (DLS.com) tissue biobank. The tissue was collected at 38 weeks gestation, flash frozen, and donated to research. The collected tissue was anonymized and de-identified and provided by a certified vendor (DLS) in compliance with established ethical standards, i.e., Health Insurance Portability and Accountability Act and the Common Rule (45 CFR 46). This type of research is exempt under the US federal regulations (45 CFR 46.102), because the use of de-identified human tissue obtained from commercial sources is classified as “not human subjects research” and is exempt from Institutional Review Board oversight.

#### Chemicals and solutions

The following solutions were used for in situ measurement of uterine myometrial contractions in acute tissue slices (C57BL/6J mice):

(S_1_) Hepes-buffered extracellular solution containing 145 mM NaCl, 5 mM KCl, 1 mM CaCl_2_, 1 mM MgSO_4_, 0.4 mM KH_2_PO_4_, and 10 mM Hepes; pH 7.3 (adjusted with NaOH); osmolarity of 300 mosmol (adjusted with glucose).

(S_2_) Oxygenated (95% O_2_, 5% CO_2_) extracellular solution containing 120 mM NaCl, 25 mM NaHCO_3_, 5 mM KCl, 1 mM CaCl_2_, 1 mM MgSO_4_, 0.4 mM KH_2_PO_4_, and 5 mM *N*,*N*-bis(2-hydroxyethyl)-2-aminoethanesulfonic acid (BES); pH 7.3 (NaOH); 300 mosmol (glucose).

(S_3_) Oxygenated (95% O_2_, 5% CO_2_) extracellular low Ca^2+^ solution containing 110 mM NaCl, 25 mM NaHCO_3_, 5 mM KCl, 1 mM MgSO_4_, 0.4 mM KH_2_PO_4_, and 5 mM BES; pH 7.3 (NaOH); 300 mosmol (glucose); [Ca^2+^]_free_ = ~10 nM (1 mM EGTA and 0.1 mM CaCl_2_). Free Ca^2+^ concentrations were calculated using WEBMAXCLITE v1.15 (RRID:SCR_000459). If not stated otherwise, chemicals were purchased from Sigma-Aldrich (Schnelldorf, Germany). VU590 (hydrochloride) was purchased from Cayman Chemicals (Ann Arbor, MI, USA). Final solvent concentrations were < 0.1%.

#### Slice preparation

Acute slices (300 to 350 μm) of uterine myometrium were prepared from pregnant mice (C57BL/6J) at day 14.5 of gestation. After removing the placenta, blocks of myometrial tissue (~5 mm by 5 mm) were embedded in 5% low-gelling temperature agarose (VWR International, Erlangen, Germany) and placed in oxygenated S_3_ (RT), and slices (300 to 350 μm) were cut on a VT1000S vibrating blade microtome (0.15 mm/s, 73 Hz; RRID:SCR_016495; Leica Biosystems, Nussloch, Germany). Slices were transferred to a submerged and oxygenated storage chamber with circulating S_2_ (RT) until use.

#### Stimulation

For stimulation, solutions and agents were applied from air pressure-driven reservoirs via an eight-in-one Ø 100-μm multibarrel “perfusion pencil” (AutoMate Scientific, Berkeley, CA, USA). Changes in focal superfusion (*57*) were software-controlled and synchronized with data acquisition by Transistor-Transistor Logic (TTL) input to 12-V dc solenoid valves using a TIB 14S digital output trigger interface (HEKA Elektronik, Lambrecht/Pfalz, Germany). Progesterone was applied simultaneously via both the bath and perfusion pencil. We routinely switched between control valves (S_1_ versus S_1_) during experiments to control for mechanical/motion artifacts.

#### Reflective light imaging

To record contractions in acute myometrial slices (C57BL/6N mice), sections were transferred to a recording chamber, and images were taken using an upright fixed-stage microscope (Leica DMI6000FS, Leica Microsystems) equipped with a charge-coupled device camera (DFC365 FX, Leica Microsystems) and Leica LAS X imaging software. Data were acquired using either a 10× (10×/0.3 HC APO L U-V-I, Leica Microsystems) or a 25× (HCX IRAPO L25X/0.95 W, Leica Microsystems) water immersion objective. When using the latter objective, magnification was adjusted to 8.75× using ×0.35 “magnification.” Following recently established protocols (*58*), we recorded bright-field or “pseudo–bright-field” reflected-light images at 2-Hz frame rate.

#### Placental pericyte isolation and primary cell culture

The uterine horn was dissected out and placed into a 10-cm dish with ice cold DPBS (without Ca^2+^ and Mg^2+^ (Thermo Fisher Scientific, 14190144)]. The placenta was then dissected away from the fetus and uterine tissue. The bottom third of the placenta was sliced off and digested in Pronase (0.1 mg/ml in DPBS; Sigma-Aldrich, 10165921001) at 37°C for 30 min in 5% CO_2_ atmosphere. Tissue was vigorously pipetted up and down every 10 min. The reaction was inhibited by adding a 5× volume of fresh growth medium (DMEM supplemented with 10% FBS). The remaining tissue was allowed to sediment for 30 s after which the supernatant was transferred into a fresh 15-ml conical tube. The placental cells were collected by centrifugation at 500*g* for 5 min, resuspended in fresh growth medium and plated on 5-mm glass coverslip in a four-well plate. The cells were allowed to attach to the coverslip for 2 hours at 37°C and 5% CO_2_ before electrophysiology measurements.

#### Human Kir7.1 cloning and recombinant expression

hACTB-RFP-Kir7.1 was a gift from B. Pattnaik (Addgene, plasmid no. 107179; http;//n2t.net/addgene:107179; RRID: Addgene_107179). Kir7.1 was cloned out of hACTB-RFP-Kir7.1 and inserted into a plasmid Internal Ribosome Entry Site (pIRES)–enhanced green fluorescent protein (EGFP) expression plasmid (pIRES-EGFP; Clontech, 6029-1) using Gibson Assembly (New England Biolabs, E5510) according to the manufacturer’ instructions and the following primers: pIRES-EGFP_fwd: 5′-TCAAGCTTCGAATTCTGCAG-3′; pIRES-EGFP_rev: 5′-TAGCGCTAGCGGATCTGAC-3′; hKir7.1_fwd: 5′-CGTCAGATCCGCTAGCGCTAATGGACAGCAGTAATTGGC-3′; hKir7.1_rev: 5′-CTGCAGAATTCGAAGCTTGATTATTCTGTCA-GTCCTGTTTC-3′ (designed using NEBuilder Assembly Tool v2.3.1). For recombinant expression, cells of the HEK cell line (HEK293, American Type Culture Collection, CRL-1573) and HUtSMCs (PromoCell, C-12576) were plated in six-well plates and transfected with pIRES2-eGFP-hKir7.1 construct using Lipofectamine 2000 reagent (Life Technologies, 11668027) according to the manufacturer’s recommendation. Overexpression was verified by the presence of hKir7.1 in immunocytochemical staining and by Western blot analysis 24 hours after transfection as mentioned below. The EGFP of the pIRES2-EGFP vector was also used to visualize the transfected cells for whole-cell patch-clamp experiments.

#### Immunohistochemistry and immunocytochemistry

For staining of uterine and placental sections, female mice were euthanized after which the uteri were dissected out. The entire nonpregnant uteri, uteri at 15.5 dpc, the placentae, and the specific sections of the pregnant myometrium were placed in formalin overnight, dehydrated, and ultimately embedded in paraffin wax. The uteri were sectioned using a microtome into 5-μm-thick sections. For Kir7.1 and α-actin staining, the sections were permeated with blocking buffer containing 0.5% Triton X-100 (Fisher BioReagents, BP151-100) and 10% goat serum (Abcam, 7481) for 30 min and incubated with primary antibody in blocking buffer overnight. After washing three times in phosphate-buffered saline (PBS), the sections were incubated with secondary antibody for 1 hour, washed three times in PBS, and mounted.

For the detection of Kir7.1 in HEK293 cells and primary cells, cells were seeded on coverslips and fixed with 4% paraformaldehyde (PFA) in PBS for 10 min, washed three times with PBS, and permeated in 0.5% Triton X-100 in PBS for 15 min. Coverslips were then blocked in 5% bovine serum albumin (BSA) in PBS at RT for 1 hour and incubated with primary antibody in 1% BSA overnight in at +4°C. After washing three times in PBS, coverslips were incubated in secondary antibody for 1 hour and then mounted. The following primary antibodies were used: mouse monoclonal anti-Kir7.1 (1:300; Santa Cruz Biotechnology, sc-398810), rabbit monoclonal anti-NG2 (1:200; Abcam, ab275024), and rabbit polyclonal anti–α-SMA antibody (1:500; EMD Millipore, ABT1487). The secondary antibodies conjugated with different fluorescent dyes were used: Cy3-conjugated donkey anti-mouse (1:500; Jackson ImmunoResearch, 715-165-150) and Cy5-conjugated donkey anti-rabbit (1:500, Jackson ImmunoResearch, 711-175-152). For mounting, we used ProLong Gold antifade reagent with 4′,6-diamidino-2-phenylindole (Invitrogen, P36935). The cells were visualized using a confocal laser scanning microscopy (Olympus FluoView FV1000).

For staining of uterine myometrial tissue slices in Fig. 2, blocks of myometrial tissue (5 mm by 5 mm) were fixed with 4% (w/v) PFA in calcium/magnesium-free PBS (PBS^−/−^; pH 7.4; 1 hour; RT). Samples were washed twice (PBS^−/−^; 5 min) and embedded in 5% agarose. Next, we cut slices (100 μm) on a VT1000S vibratome (Leica Biosystems). For blocking and permeabilization, sections were incubated (1.5 hours; RT) in PBS^−/−^ containing Triton X-100 (0.3%)/BSA (3%). After washing (PBS^−/−^; three times for 15 min), sections were incubated with fluorescein isothiocyanate (FITC)–conjugated monoclonal anti-actin, anti–α-SMA antibody [1:200 in BSA (3%); 2 hours; RT; α-SMA–FITC; catalog no. F3777, MilliporeSigma]. For nuclear counterstaining, sections were then incubated in PBS^−/−^ containing DRAQ5 (1:500; 1 hour; RT; Thermo Fisher Scientific).

These fluorescent images were taken using a Leica TCS SP8 confocal microscope, equipped with a 20× immersion objective (HC PL APO 20×/0.75 IMM CORR CS2; Leica Microsystems). Digital images were uniformly adjusted for brightness and contrast using Fiji-ImageJ (RRID:SCR_002285).

#### Western blotting

To detect Kir7.1 in HEK293 cells transfected with the pIRES2-EGFP/Kir7.1 construct, the cells were cultured for 24 hours after transfection and harvested by standard procedures as reported in (*28*). To detect Kir7.1 in placenta (fig. S9), three different regions of postpartum placenta (~3 mm^3^) were excised in ice-cold radioimmunoprecipitation assay buffer containing cOmplete proteinase inhibitors (Roche) and homogenized using Benchmark Scientific D2400-R BeadBlaster Tissue Homogenizer at 4°C. The samples were analyzed by Western blotting, using the mouse monoclonal antibody against Kir7.1 (1:2500 dilution), rabbit monoclonal anti-NG2 (1:2000; Abcam, ab275024), and a peroxidase-conjugated goat anti-mouse secondary antibody (1:15,000 dilution; EMD-Millipore, AP181P), as well as a peroxidase-conjugated anti-rabbit secondary antibody. To ensure equal sample loading, the membrane was stripped by incubation with OneMinute Plus Strip (GM Biosciences, GM6011) according to the manufacturer’s instructions. Thereafter, the membrane was re-hybridized with a mouse monoclonal anti-actin antibody (1:5000 dilution; Abcam, ab3280-500) and the peroxidase-conjugated goat anti-mouse secondary antibody (1:15,000 dilution).

#### Reagents for electrophysiology

KMeSO_3_ was acquired from Alfa Aesar (39505) and CsMeSO3 from Sigma-Aldrich (C1426). Progesterone was purchased from MilliporeSigma and Tocris, 17β-estradiol from Sigma-Aldrich and Tocris, and VU590 {7,13-bis[(4-nitrophenyl)methyl]-1,4,10-trioxa-7,13-diazacyclopentadecane} dihydrochloride from Thermo Fisher Scientific, Cayman Chemicals, and Tocris. DOF (3757) was purchased from Tocris. There were no differences between action of these compounds purchased from different vendors. All other compounds were from Cayman Chemicals. For patch-clamp measurements, all bath solutions contained a concentration of 1:1000 of ethanol and dimethyl sulfoxide (used as vehicle controls), the solvents used to dissolve the steroid hormones and the antagonist VU590, respectively.

#### Electrophysiology

Cells plated on 5-mm glass coverslips were placed in a perfusion chamber (Warner Instruments, RC-24E). Kir7.1 current was recorded following the procedures previously described in (*59*). Specifically, data were acquired using the Clampex 10.5 software (Molecular Devices), which controlled an AxoPatch 200B amplifier and an Axon Digidata 1550A digitizer (both Molecular Devices) with integrated Humbug noise eliminator. Gigaohm seals were established in Krebs Ringer Solution [135 mM NaCl, 5 mM KCl, 1 mM MgSO_4_, 0.4 mM K_2_HPO_4_, 5.5 mM glucose, 20 mM Hepes, and 1 mM CaCl_2_ (pH 7.3, 310 mosmol/liter)]. The patch pipette was filled with a Cs^+^-based solution [130 mM CsMeSO_3_, 20 mM Hepes, 5 mM 1,2-bis(2-aminophenoxy)ethane-*N*,*N*,*N*′,*N*′-tetraacetic acid, and 1 mM MgCl_2_ (pH 7.3 adjusted with CsOH, 295 mosmol/liter)] and had a resistance of 4 to 7 megohms. The bath solutions contained 130 mM KMeSO_3_, 43 mM Hepes, and 1 mM MgCl_2_ (pH 7.3, 310 mosmol/L). All buffers, including those containing steroid hormones, were applied under constant perfusion. Measurements were performed at 10 kHz and filtered at 1 kHz. Cells were stimulated every 5 s by voltage ramps from −80 to +80 mV with a holding potential of 0 mV. Access resistance and membrane capacitance were within 8 to 13 megohms and 4.2 ± 0.66 pF for pericytes, 7 to 13 megohms and 14.2 ± 0.5 pF for HEK293 cells, 8 to 18 megohms and 12.7 ± 2.2 pF for murine myocytes, and 10 to 18 megohms and 19.7 ± 2.4 pF for human myocytes. Membrane capacitance served as a proxy for the cell surface area and, thus, for normalization of current amplitudes (i.e. current density). Capacitance artifacts were graphically removed in OriginPro 8.6 (OriginLab Corp.).

#### Data analysis

All data were obtained from independent experiments performed on at least 3 days. Individual numbers of regions of interest (ROIs) and/or experiments (*n*) are denoted in figures and/or captions. Statistical analyses were performed using Kruskal-Wallis tests with Tukey’s post hoc test. Tests and corresponding *P* values that report statistical significance (≤0.05) are individually specified in captions.

Quantitative analysis from time-lapse reflected-light image series was performed as previously described (*58*) with minor adjustments. Briefly, for each frame at a given time point *t*_*i*_, the registration algorithm computed a flow or displacement vector field

$$V_{i}=\left(v_{1,1}\ldots v_{1,n}\ldots \ldots \ldots v_{m,1}\ldots v_{m,n}\right)$$

where $v_{1,1}=\left(x,y\right)$ is a vector indicating strength and direction of pixel displacement (1, 1) between time points *t*_0_ and *t*_*i*_. The average norm $\left|V_{i}\right|=\frac{1}{\mathrm{mn}}\sum_{p,q}\left\|v_{p,q}\right\|$ is a measure for the effort that is necessary to register the image at *t*_0_ to the image at *t*_*i*_. We quantified the flow change *c*_*i*_ = *s*_*i*_ − *s*_*i*−1_ as the change of flow strength between two consecutive time points/frames. Here, *s*_*i*_ values were preprocessed by smoothing with a moving average filter.

Next, we assessed both the speed and direction of movement on the basis of cumulative changes in *x* and *y* coordinates. Linear regression derived a slope (a) that represents the relationship between both dimensions. For standardization and quantification, we calculated a direction vector (**X**, **Y**). Its length (*L*) was obtained by computing the square root of (*a*^2^ + 1). Combining vector normalization (i.e., **X** = 1/*L*; **Y** = *a*/*L*) with *x* and *y* coordinate shifts enabled us to compute the projected velocity: *x* * **X** + *y* * **Y**; measuring directed velocity in a two-dimensional space.

Movies were cropped to ensure that frame 1 represents the tissue in a relaxed state. ROIs were manually defined. All positive values were normalized to the maximum, while all negative values were normalized to the minimum. When plotted as a function of time, the resulting trace exhibits specific waveform shapes corresponding to contractions. These waveforms are characterized by a sharp increase, a positive peak, followed by rebound in opposite direction, and a negative peak. For semiautomatic unbiased identification of contraction-relaxation events by corresponding positive and negative peaks, we used an iterative peak detection approach that uses both positive and negative thresholding and subsequent “binary” classification [as either positive (+1) or negative (−1) peaks]. Initially, signals surpassing 35% of the maximum or minimum were classified as positive or negative peaks. These peaks are registered and subsequently erased from the trace to prevent subsequent (re)registration by the algorithm. In a second iteration, the residual trace is analyzed again on the basis of a 25% peak detection threshold. An additional iteration (15% threshold) failed to uncover any remaining peaks. After iterative peak detection, the signal is represented by a trace that fluctuates between three states (i.e., +1, 0, and −1). The first derivative of this trace marks the start and end points of individual peaks and is used to identify peak sequences that represent the specific waveform shapes corresponding to contraction-relaxation events (i.e., [1, −1, −1, 1] or [−1, 1, 1, −1]). Next, events were filtered according to duration criteria (>15 s; <30 s). Last, event labels are matched to the original raw trace.

The algorithm achieved 85% sensitivity and 90% positive predictive value in detecting contraction-relaxation events. The corresponding Python code is found at https://zenodo.org/records/14161457.

For statistical analyses of the activation and inhibition of Kir7.1 by different steroid hormones and the antagonists VU590 and ML418, the Clampfit 10.3 (Molecular Devices), OriginPro 8.6 (OriginLab), and GraphPad Prism 5 (GraphPad Software) software were used. Unpaired and paired *t* tests were used to determine statistical significance, assigning *P* ≤ 0.05 as the limit. All results are shown with SEM.

## References

[1] What is the strongest muscle in the human body (2019/updated 2024); www.loc.gov/everyday-mysteries/biology-and-human-anatomy/item/what-is-the-strongest-muscle-in-the-human-body/.

[2] S. Wray, S. Arrowsmith, Uterine excitability and ion channels and their changes with gestation and hormonal environment. Annu. Rev. Physiol. 83, 331–357 (2021). 33158376 10.1146/annurev-physiol-032420-035509

[3] S. Wray, K. Noble, Sex hormones and excitation-contraction coupling in the uterus: The effects of oestrous and hormones. J. Neuroendocrinol. 20, 451–461 (2008). 18266942 10.1111/j.1365-2826.2008.01665.x

[4] F. S. Khan-Dawood, M. Y. Dawood, Estrogen and progesterone receptor and hormone levels in human myometrium and placenta in term pregnancy. Am. J. Obstet. Gynecol. 150, 501–505 (1984). 6496580 10.1016/s0002-9378(84)90428-9

[5] S. Astle, D. M. Slater, S. Thornton, The involvement of progesterone in the onset of human labour. Eur. J. Obstet. Gynecol. Reprod. Biol. 108, 177–181 (2003). 12781407 10.1016/s0301-2115(02)00422-0

[6] E. Hamburg-Shields, S. Mesiano, The hormonal control of parturition. Physiol. Rev. 104, 1121–1145 (2024). 38329421 10.1152/physrev.00019.2023 PMC11380996

[7] S. A. M. Jerome, F. Strauss III, “Placental production of peptide, steroid, and lipid hormones” in *Maternal-Fetal and Neonatal Endocrinology* (Academic Press, 2019), chap. 41.

[8] B. V. Natale, P. Mehta, P. Vu, C. Schweitzer, K. Gustin, R. Kotadia, D. R. C. Natale, Reduced uteroplacental perfusion pressure (RUPP) causes altered trophoblast differentiation and pericyte reduction in the mouse placenta labyrinth. Sci. Rep. 8, 17162 (2018). 30464252 10.1038/s41598-018-35606-x PMC6249310

[9] A. Csapo, Progesterone block. Am. J. Anat. 98, 273–291 (1956). 13326855 10.1002/aja.1000980206

[10] O. Shynlova, L. Nadeem, J. Zhang, C. Dunk, S. Lye, Myometrial activation: Novel concepts underlying labor. Placenta 92, 28–36 (2020). 32056784 10.1016/j.placenta.2020.02.005

[11] M. Perusquia, Nongenomic action of steroids in myometrial contractility. Endocrine 15, 063–070 (2001). 10.1385/ENDO:15:1:063 11572328

[12] A. A. Merlino, T. N. Welsh, H. Tan, L. J. Yi, V. Cannon, B. M. Mercer, S. Mesiano, Nuclear progesterone receptors in the human pregnancy myometrium: evidence that parturition involves functional progesterone withdrawal mediated by increased expression of progesterone receptor-A. J. Clin. Endocrinol. Metab. 92, 1927–1933 (2007). 17341556 10.1210/jc.2007-0077

[13] F. Hertelendy, T. Zakar, Regulation of myometrial smooth muscle functions. Curr. Pharm. Des. 10, 2499–2517 (2004). 15320759 10.2174/1381612043383926

[14] N. E. Renthal, K. C. Williams, A. P. Montalbano, C. C. Chen, L. Gao, C. R. Mendelson, Molecular regulation of parturition: A myometrial perspective. Cold Spring Harb. Perspect. Med. 5, a023069 (2015). 26337112 10.1101/cshperspect.a023069 PMC4632865

[15] S. Mesiano, Progesterone withdrawal and parturition. J. Steroid Biochem. Mol. Biol. 224, 106177 (2022). 36096351 10.1016/j.jsbmb.2022.106177

[16] C. V. Bishop, F. Stormshak, Nongenomic action of progesterone inhibits oxytocin-induced phosphoinositide hydrolysis and prostaglandin F_2α_ secretion in the ovine endometrium. Endocrinology 147, 937–942 (2006). 16254031 10.1210/en.2005-0869

[17] C. McCloskey, C. Rada, E. Bailey, S. McCavera, H. A. van den Berg, J. Atia, D. A. Rand, A. Shmygol, Y. W. Chan, S. Quenby, J. J. Brosens, M. Vatish, J. Zhang, J. S. Denton, M. J. Taggart, C. Kettleborough, D. Tickle, J. Jerman, P. Wright, T. Dale, S. Kanumilli, D. J. Trezise, S. Thornton, P. Brown, R. Catalano, N. Lin, S. K. England, A. M. Blanks, The inwardly rectifying K^+^ channel KIR7.1 controls uterine excitability throughout pregnancy. EMBO Mol. Med. 6, 1161–1174 (2014). 25056913 10.15252/emmm.201403944 PMC4197863

[18] M. Perusquia, J. Jasso-Kamel, Influence of 5α - and 5β -reduced progestins on the contractility of isolated human myometrium at term. Life Sci. 68, 2933–2944 (2001). 11411793 10.1016/s0024-3205(01)01089-x

[19] V. Tskhay, A. Schindler, M. Shestakova, O. Klimova, A. Narkevich, The role of progestogen supplementation (dydrogesterone) in the prevention of preeclampsia. Gynecol. Endocrinol. 36, 698–701 (2020). 31876197 10.1080/09513590.2019.1706085

[20] M. B. Sammour, H. El-Kabarity, M. M. Fawzy, A. E. Schindler, Prevention and treatment of pregnancy-induced hypertension (preeclampsia) with progestogens. J. Steroid Biochem. Mol. Biol. 97, 439–440 (2005). 16236493 10.1016/j.jsbmb.2005.08.014

[21] F. Cadepond, A. Ulmann, E. E. Baulieu, RU486 (mifepristone): Mechanisms of action and clinical uses. Annu. Rev. Med. 48, 129–156 (1997). 9046951 10.1146/annurev.med.48.1.129

[22] O. Shynlova, P. Tsui, A. Dorogin, M. Chow, S. J. Lye, Expression and localization of alpha-smooth muscle and gamma-actins in the pregnant rat myometrium. Biol. Reprod. 73, 773–780 (2005). 15972885 10.1095/biolreprod.105.040006

[23] T. Kawarabayashi, J. M. Marshall, Factors influencing circular muscle activity in the pregnant rat uterus. Biol. Reprod. 24, 373–379 (1981). 7213881 10.1095/biolreprod24.2.373

[24] M. J. Berridge, Smooth muscle cell calcium activation mechanisms. J. Physiol. 586, 5047–5061 (2008). 18787034 10.1113/jphysiol.2008.160440 PMC2652144

[25] M. Kumar, B. R. Pattnaik, Focus on Kir7.1: physiology and channelopathy. Channels (Austin) 8, 488–495 (2014). 25558901 10.4161/19336950.2014.959809 PMC4594557

[26] G. Krapivinsky, I. Medina, L. Eng, L. Krapivinsky, Y. Yang, D. E. Clapham, A novel inward rectifier K^+^ channel with unique pore properties. Neuron 20, 995–1005 (1998). 9620703 10.1016/s0896-6273(00)80480-8

[27] W. Yin, H. T. Kim, S. P. Wang, F. Gunawan, L. Wang, K. Kishimoto, H. Zhong, D. Roman, J. Preussner, S. Guenther, V. Graef, C. Buettner, B. Grohmann, M. Looso, M. Morimoto, G. Mardon, S. Offermanns, D. Y. R. Stainier, The potassium channel KCNJ13 is essential for smooth muscle cytoskeletal organization during mouse tracheal tubulogenesis. Nat. Commun. 9, 2815 (2018). 30022023 10.1038/s41467-018-05043-5 PMC6052067

[28] I. Björkgren, S. Mendoza, D. H. Chung, M. Haoui, N. T. Petersen, P. V. Lishko, The epithelial potassium channel Kir7.1 is stimulated by progesterone. J. Gen. Physiol. 153, e202112924 (2021). 34387656 10.1085/jgp.202112924 PMC8374857

[29] M. Haoui, N. T. Petersen, I. Björkgren, D. H. Chung, P. V. Lishko, Choroid plexus epithelial cells as a model to study nongenomic steroid signaling and its effect on ion channel function. Methods Enzymol. 654, 297–314 (2021). 34120718 10.1016/bs.mie.2021.03.004

[30] R. M. Brenner, N. B. West, Hormonal regulation of the reproductive tract in female mammals. Annu. Rev. Physiol. 37, 273–302 (1975). 164819 10.1146/annurev.ph.37.030175.001421

[31] D. R. Swale, H. Kurata, S. V. Kharade, J. Sheehan, R. Raphemot, K. R. Voigtritter, E. E. Figueroa, J. Meiler, A. L. Blobaum, C. W. Lindsley, C. R. Hopkins, J. S. Denton, ML418: The first selective, sub-micromolar pore blocker of Kir7.1 potassium channels. ACS Chem. Nerosci. 7, 1013–1023 (2016). 10.1021/acschemneuro.6b00111 PMC5131535 27184474

[32] S. Monticone, R. J. Auchus, W. E. Rainey, Adrenal disorders in pregnancy. Nat. Rev. Endocrinol. 8, 668–678 (2012). 22965163 10.1038/nrendo.2012.155

[33] S. Mesiano, T. N. Welsh, Steroid hormone control of myometrial contractility and parturition. Semin. Cell Dev. Biol. 18, 321–331 (2007). 17613262 10.1016/j.semcdb.2007.05.003

[34] C. V. Bishop, Progesterone inhibition of oxytocin signaling in endometrium. Front. Neurosci. 7, 138 (2013). 23966904 10.3389/fnins.2013.00138 PMC3735988

[35] S. Gupta, A. S. Roman, 17-α hydroxyprogesterone caproate for the prevention of preterm birth. Womens Health (Lond) 8, 21–30 (2012). 22171770 10.2217/whe.11.78

[36] P. J. Meis, M. Klebanoff, E. Thom, M. P. Dombrowski, B. Sibai, A. H. Moawad, C. Y. Spong, J. C. Hauth, M. Miodovnik, M. W. Varner, K. J. Leveno, S. N. Caritis, J. D. Iams, R. J. Wapner, D. Conway, M. J. O’Sullivan, M. Carpenter, B. Mercer, S. M. Ramin, J. M. Thorp, A. M. Peaceman, S. Gabbe, Prevention of recurrent preterm delivery by 17 alpha-hydroxyprogesterone caproate. N. Engl. J. Med. 348, 2379–2385 (2003). 12802023 10.1056/NEJMoa035140

[37] S. J. Choi, Use of progesterone supplement therapy for prevention of preterm birth: review of literatures. Obstet. Gynecol. Sci. 60, 405–420 (2017). 28989916 10.5468/ogs.2017.60.5.405 PMC5621069

[38] K. Yasuda, G. I. Sumi, H. Murata, N. Kida, T. Kido, H. Okada, The steroid hormone dydrogesterone inhibits myometrial contraction independently of the progesterone/progesterone receptor pathway. Life Sci. 207, 508–515 (2018). 29981319 10.1016/j.lfs.2018.07.004

[39] A. E. Schindler, Progestational effects of dydrogesterone in vitro, in vivo and on the human endometrium. Maturitas 65, S3–S11 (2009). 19969432 10.1016/j.maturitas.2009.10.011

[40] F. G. Mirza, A. Patki, C. Pexman-Fieth, Dydrogesterone use in early pregnancy. Gynecol. Endocrinol. 32, 97–106 (2016). 26800266 10.3109/09513590.2015.1121982

[41] M. Bygdeman, K. Gemzell Danielsson, L. Marions, M. Swahn, Pregnancy termination. Steroids 65, 801–805 (2000). 11108891 10.1016/s0039-128x(00)00182-3

[42] D. W. Matt, G. J. Macdonald, Placental steroid production by the basal and labyrinth zones during the latter third of gestation in the rat. Biol. Reprod. 32, 969–977 (1985). 4005353 10.1095/biolreprod32.4.969

[43] R. Ohlsson, P. Falck, M. Hellström, P. Lindahl, H. Boström, G. Franklin, L. Ahrlund-Richter, J. Pollard, P. Soriano, C. Betsholtz, PDGFB regulates the development of the labyrinthine layer of the mouse fetal placenta. Dev. Biol. 212, 124–136 (1999). 10419690 10.1006/dbio.1999.9306

[44] T. Yamazaki, Y. S. Mukouyama, Tissue specific origin, development, and pathological perspectives of pericytes. Front. Cardiovasc. Med. 5, 78 (2018). 29998128 10.3389/fcvm.2018.00078 PMC6030356

[45] N. B. Hamilton, D. Attwell, C. N. Hall, Pericyte-mediated regulation of capillary diameter: A component of neurovascular coupling in health and disease. Front. Neuroenergetics 2, 5 (2010). 20725515 10.3389/fnene.2010.00005 PMC2912025

[46] March of Dimes (2024); www.marchofdimes.org/find-support/topics/pregnancy/preeclampsia.

[47] Centers for Disease Control and Prevention (CDC), Maternal infant health, Preterm birth (CDC, 2024); www.cdc.gov/maternal-infant-health/preterm-birth/.

[48] N. J. Waitzman, A. Jalali, S. D. Grosse, Preterm birth lifetime costs in the United States in 2016: An update. Semin. Perinatol. 45, 151390 (2021). 33541716 10.1016/j.semperi.2021.151390 PMC10549985

[49] EPPPIC Group, Evaluating Progestogens for Preventing Preterm birth International Collaborative (EPPPIC): Meta-analysis of individual participant data from randomised controlled trials. Lancet 397, 1183–1194 (2021). 33773630 10.1016/S0140-6736(21)00217-8

[50] Food and Drug Administration (FDA), FDA commissioner and chief scientist announce decision to withdraw approval of Makena (FDA, 2023); www.fda.gov/news-events/press-announcements/fda-commissioner-and-chief-scientist-announce-decision-withdraw-approval-makena.

[51] M. Hines, K. A. Lyseng-Williamson, E. D. Deeks, 17 α-Hydroxyprogesterone caproate (Makena): A guide to its use in the prevention of preterm birth. Clin. Drug Investig. 33, 223–227 (2013). 10.1007/s40261-013-0060-6 23413110

[52] S. Mesiano, E. C. Chan, J. T. Fitter, K. Kwek, G. Yeo, R. Smith, Progesterone withdrawal and estrogen activation in human parturition are coordinated by progesterone receptor A expression in the myometrium. J. Clin. Endocrinol. Metab. 87, 2924–2930 (2002). 12050275 10.1210/jcem.87.6.8609

[53] H. Schock, A. Zeleniuch-Jacquotte, E. Lundin, K. Grankvist, H. A. Lakso, A. Idahl, M. Lehtinen, H. M. Surcel, R. T. Fortner, Hormone concentrations throughout uncomplicated pregnancies: A longitudinal study. BMC Pregnancy Childbirth 16, 146 (2016). 27377060 10.1186/s12884-016-0937-5 PMC4932669

[54] Food and Drug Administration (FDA), Mifepristone U.S. post-marketing adverse events summary through 12/31/2022 (2022); www.fda.gov/media/164331/download.

[55] G. Ghosh, J. Grewal, T. Männistö, P. Mendola, Z. Chen, Y. Xie, K. S. Laughon, Racial/ethnic differences in pregnancy-related hypertensive disease in nulliparous women. Ethn. Dis. 24, 283–289 (2014). 25065068 PMC4171100

[56] Protein Atlas (2024).

[57] S. Veitinger, T. Veitinger, S. Cainarca, D. Fluegge, C. H. Engelhardt, S. Lohmer, H. Hatt, S. Corazza, J. Spehr, E. M. Nauhaus, M. Spehr, Purinergic signalling mobilizes mitochondrial Ca^2+^ in mouse Sertoli cells. J. Physiol. 589, 5033–5055 (2011). 21859825 10.1113/jphysiol.2011.216309 PMC3225664

[58] D. Fleck, L. Kenzler, N. Mundt, M. Strauch, N. Uesaka, R. Moosmann, F. Bruentgens, A. Missel, A. Mayerhofer, D. Merhof, J. Spehr, M. Spehr, ATP activation of peritubular cells drives testicular sperm transport. eLife 10, e62885 (2021). 33502316 10.7554/eLife.62885 PMC7840184

[59] M. Haoui, thesis, University of California, Berkeley, CA (2020); https://escholarship.org/uc/item/5d8744ks.

