# Supplementary material for: Kir7.1 is the physiological target for hormones and steroids that regulate uteroplacental function
Source: Sci Adv. 2025 Mar 5;11(10):eadr5086. doi: 10.1126/sciadv.adr5086 (PMC11881918; doi:10.1126/sciadv.adr5086)
Supplement: Supplementary file 1 — Figs. S1 to S9 Legends for movies S1 to S3 [file sciadv.adr5086_sm.pdf]

Science Advances
AAAS

Supplementary Materials for

Kir7.1 is the physiological target for hormones and steroids that regulate uteroplacental function

Monika Haoui *et al.*

Corresponding author: Polina V. Lishko, lishko@wustl.edu; Marc Spehr, m.spehr@sensorik.rwth-aachen.de

Sci. Adv. **11**, eadr5086 (2025)
DOI: 10.1126/sciadv.adr5086

The PDF file includes:

Figs. S1 to S9
Legends for movies S1 to S3

Other Supplementary Material for this manuscript includes the following:

Movies S1 to S3

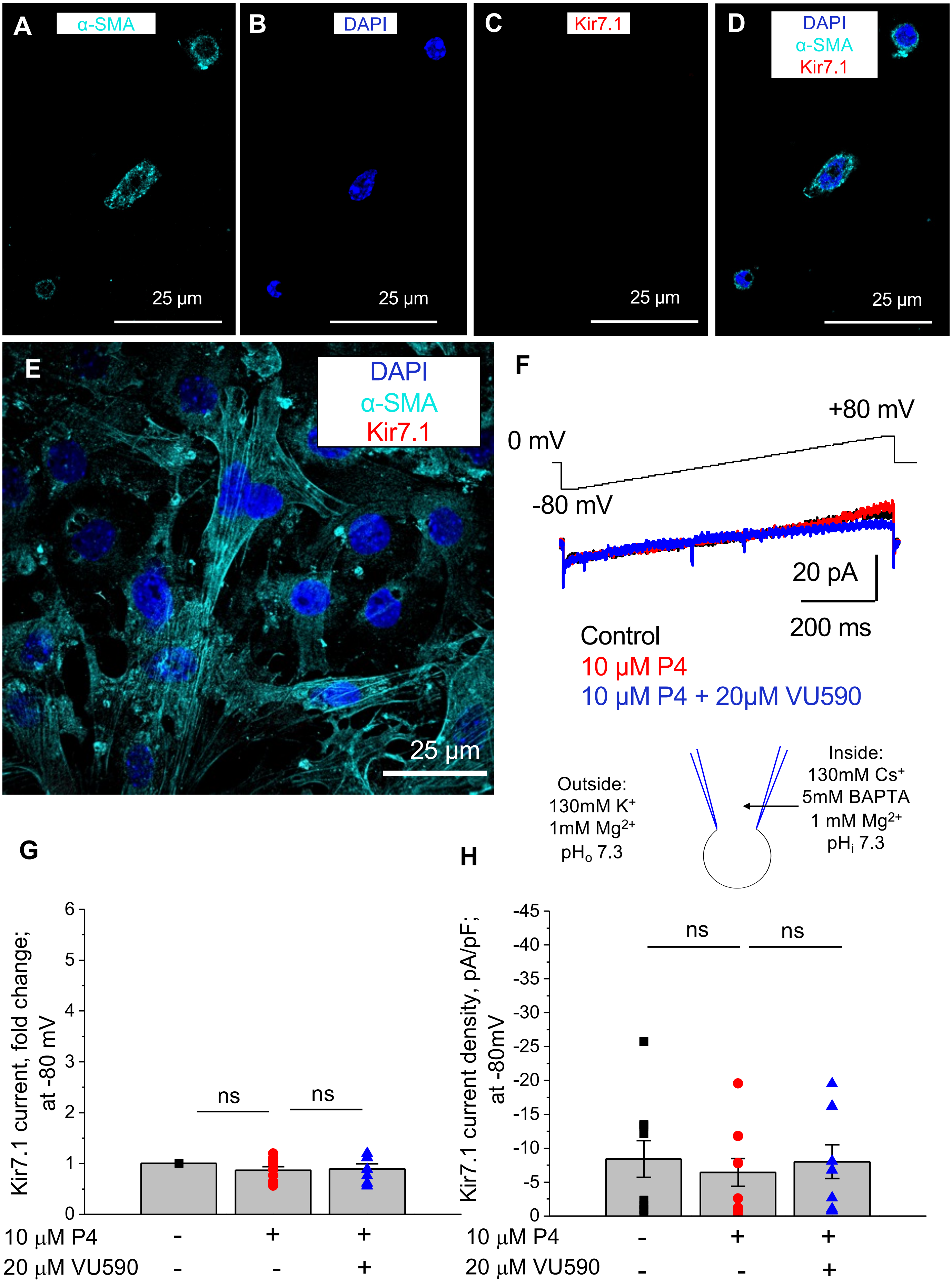

A
α-SMA
25 μm
B
DAPI
25 μm
C
Kir7.1
25 μm
D
DAPI
α-SMA
Kir7.1
25 μm
E
DAPI
α-SMA
Kir7.1
25 μm
F
+80 mV
0 mV
-80 mV
20 pA
200 ms
Control
10 μM P4
10 μM P4 + 20μM VU590
Outside:
130mM K+
1mM Mg2+
pHo 7.3
Inside:
130mM Cs+
5mM BAPTA
1 mM Mg2+
pHi 7.3
G
Kir7.1 current, fold change; at -80 mV
ns
ns
10 μM P4 - + +
20 μM VU590 - - +
H
Kir7.1 current density, pA/pF; at -80mV
ns
ns
10 μM P4 - + +
20 μM VU590 - - +

Figure S1

Supplemental Figure 1. Kir7.1 recordings from nonpregnant murine uteri. A-D. Same day-isolated myocytes from the nonpregnant uterus that were assessed using electrophysiology as shown below in (F-H). Myocytes were co-stained with anti-smooth muscle alpha-actin antibody (α-SMA; cyan), anti-Kir7.1 antibody (Kir7.1; red) and DAPI (blue, indicating nuclei). Lack of red signal in (C) indicates absence of Kir7.1 expression. **E.** Primary culture of isolated myocytes from the nonpregnant uteri in culture for 24 hours after isolation. The cells were identified by α-SMA antibody (cyan), probed with anti-Kir7.1 antibody (red) and DAPI (blue). No Kir7.1-positive staining was observed. **F.** Representative residual potassium currents recorded from myocytes isolated from uteri of 3-month-old nonpregnant mice in response to a voltage ramp as indicated from a holding potential of 0 mV. Recordings reveal the lack of Kir7.1 activity. The conductance recorded under control condition (i.e. absence of P4) is similar to conductances recorded in presence of 10 μM P4 or 10 μM P4 with 20 μM VU590. **G&H.** Bar charts displaying the average fold change in current (G) and the corresponding current densities (H) of Kir7.1 at -80 mV recorded from isolated myocytes from nonpregnant uteri (voltage ramp recording as in (F) collected from at least three different mice). Statistics for (G) are as follows: (control): n=10; P4: 0.86 ± 0.08, n=9; (P4 + VU590): 0.89 ± 0.01, n=7. Amplitudes were normalized to control. Statistics for (H) are as follows: (control): -8.40 ± 2.69, n=10; (P4): -6.43 ± 2.05, n=10; (P4 + VU590): -8.01 ± 2.49, n=9. Statistical significance was calculated using nonparametric Kruskall-Wallis test, n.s. stands for non-significant. Data are means ± SEM.

Figure S2

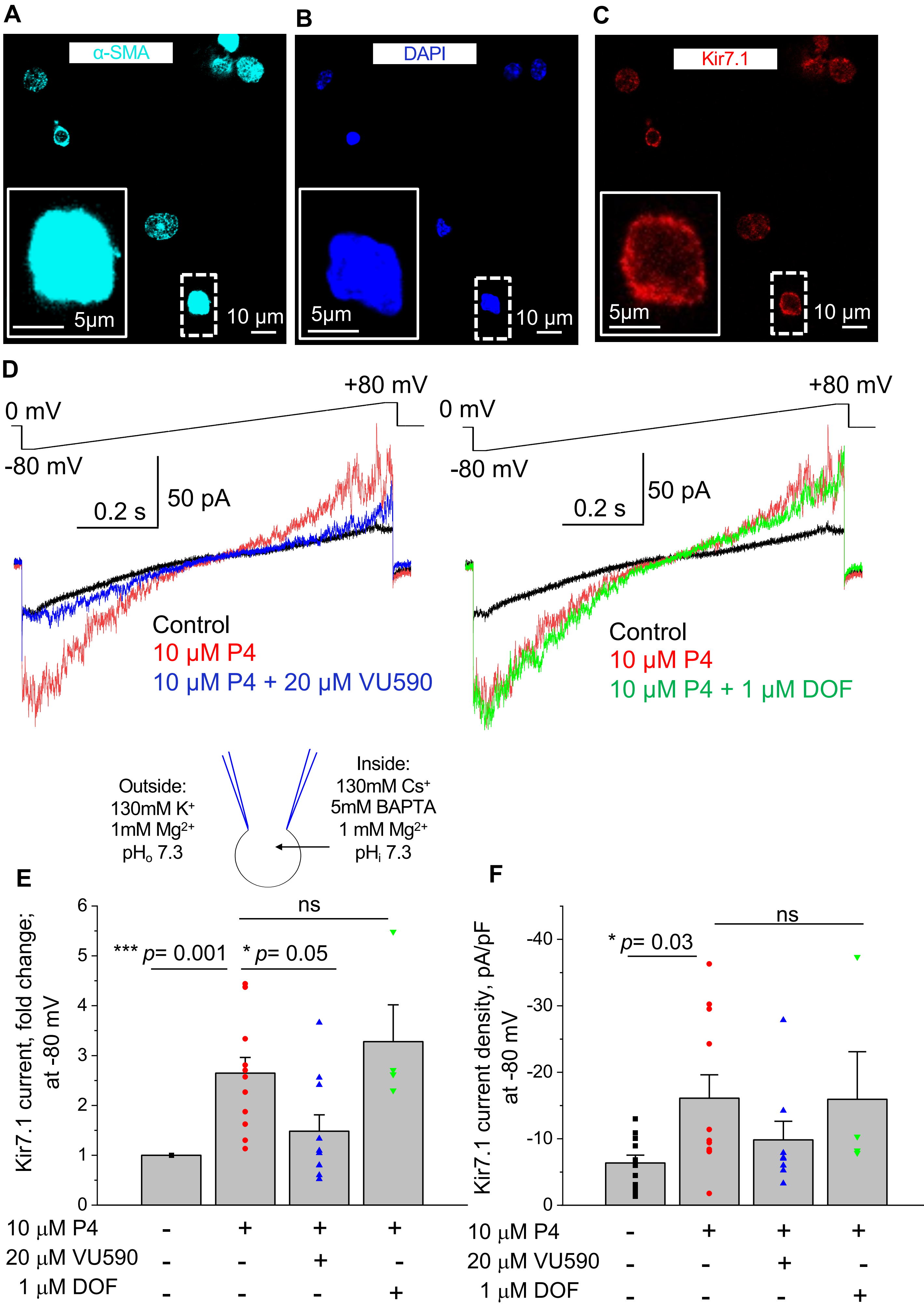
A
α-SMA
5μm
10 μm
B
DAPI
5μm
10 μm
C
Kir7.1
5μm
10 μm
D
0 mV
+80 mV
-80 mV
0.2 s
50 pA
Control
10 μM P4
10 μM P4 + 20 μM VU590
0 mV
+80 mV
-80 mV
0.2 s
50 pA
Control
10 μM P4
10 μM P4 + 1 μM DOF
Outside:
130mM K+
1mM Mg2+
pHo 7.3
Inside:
130mM Cs+
5mM BAPTA
1 mM Mg2+
pHi 7.3
E
Kir7.1 current, fold change; at -80 mV
*** p= 0.001
* p= 0.05
ns
10 μM P4 - + + +
20 μM VU590 - - + -
1 μM DOF - - - +
F
Kir7.1 current density, pA/pF at -80 mV
* p= 0.03
ns
10 μM P4 - + + +
20 μM VU590 - - + -
1 μM DOF - - - +

Supplemental Figure 2. Kir7.1 recordings from uteri of pregnant mice. A-C. Same day primary culture of isolated myocytes from 15.5 dpc uterus show α-SMA (cyan), as well as Kir7.1 presence (anti-Kir7.1 antibody; red). Nuclei were visualized by DAPI (blue). Insert shows the same zoomed-in myocyte. **D.** Representative traces recorded from isolated myocytes from 15.5 dpc uteri of 3-month-old mice in response to an indicated voltage ramp from a holding potential of 0 mV. Left panel: representative response from a subpopulation of cells exhibiting strong response to P4 that was abolished by an exposure to VU590. Right panel: Application of ERG channel inhibitor, dofetilide (DOF), didn't affect P4 potentiation, indicating the absence of ERG involvement in this effect. **E&F.** The average current fold change (E) and the corresponding current densities (F) of Kir7.1 recorded at -80 mV from a combined population of murine myocytes, isolated from 15.5 dpc uteri. Statistics for (E) are as follows: (control): n=12; (P4): 2.65 ± 0.32, n=12; (P4 + VU590): 1.48 ± 0.33, n=10; (P4 + DOF): 3.28 ± 0.74, n=4. Amplitudes were normalized to control. Statistics for (F; pA/pF) are as follows: (control): -6.35 ± 1.18, n=12; (P4): -16.12 ± 3.51, n= 12; (P4 + VU590): -9.83 ± 2.81, n=8; (P4 + DOF): -15.94 ± 7.16, n= 4. Statistical significance was calculated using the paired t-test, and the significance of changes is indicated as follows: ***, $p \leq 0.001$; *, $p \leq 0.05$; Data are means ± SEM.

Figure S3

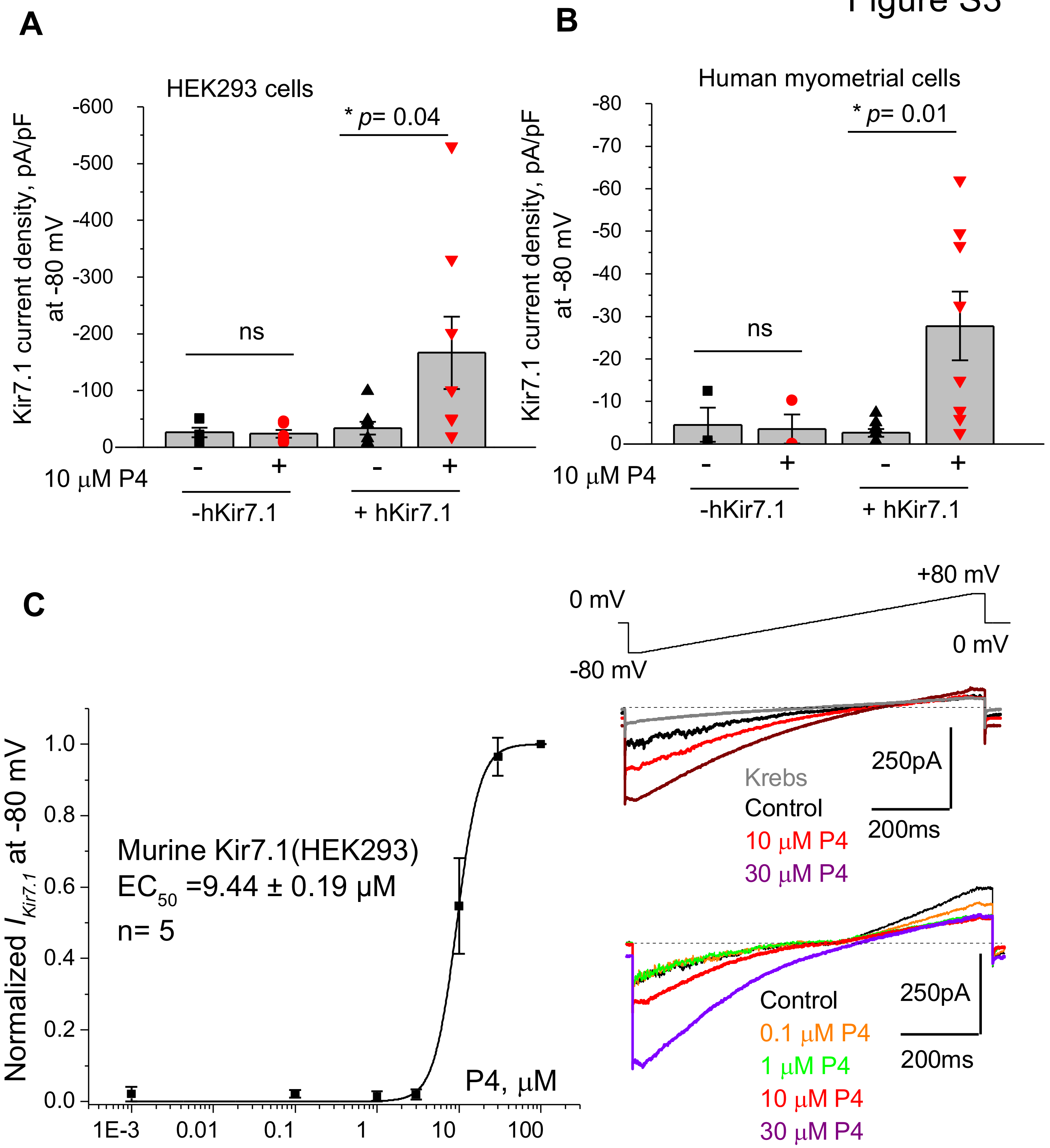
A
HEK293 cells
Kir7.1 current density, pA/pF at -80 mV
-600
-500
-400
-300
-200
-100
0
* p= 0.04
ns
10 μM P4
-
+
-
+
-hKir7.1
+ hKir7.1
B
Human myometrial cells
Kir7.1 current density, pA/pF at -80 mV
-80
-70
-60
-50
-40
-30
-20
-10
0
* p= 0.01
ns
10 μM P4
-
+
-
+
-hKir7.1
+ hKir7.1
C
Normalized I Kir7.1 at -80 mV
1.0
0.8
0.6
0.4
0.2
0.0
Murine Kir7.1(HEK293)
EC50 =9.44 ± 0.19 μM
n= 5
P4, μM
1E-3
0.01
0.1
1
10
100
+80 mV
0 mV
0 mV
-80 mV
Krebs
Control
10 μM P4
30 μM P4
250pA
200ms
Control
0.1 μM P4
1 μM P4
10 μM P4
30 μM P4
250pA
200ms

Supplemental Figure 3. Progesterone activates both mouse and human recombinant Kir7.1. A. The average current densities of hKir7.1 transiently expressed in HEK293 cells obtained at -80 mV and recorded as depicted in Fig 3E. **B.** The average current densities of hKir7.1 transiently expressed in human myometrial cells and recorded at -80 mV as depicted in Fig. 3F. Statistics for (A) are as follows: (i) HEK293 transfected with empty vector pIRES2-eGFP; (control): -25.91 ± 8.30, n=6; (P4): -23.50 ± 6.86, n=6; (ii) HEK293-hKir7.1; (control):-33.51 ± 11.39, n=8; (P4): -166.61 ± 63.74, n=8. Statistics for (B) are as follows: (i) nontransfected HUtSMC; (control): -4.51 ± 3.98, n=8; (P4): -3.50 ± 3.40, n= 8; (ii) HUtSMC-hKir7.1; (control): -2.63 ± 0.90, n=8; (P4): -27.7 ± 8.12, n=8. Statistical significance was calculated using the paired t-test, and the significance of changes is indicated as follows: *, $p \leq 0.05$; n.s. stands for non-significant. Data are means ± SEM. **C.** Dose dependence of potentiation of potassium inward $I_{Kir7.1}$ by P4 recorded from HEK293 cells after 24-hour transfection with pIRES2-eGFP-mKir7 and exposed to varying concentrations of P4. Current amplitudes were measured at −80 mV by averaging 3 to 5 original current traces and normalized to maximal response to P4. Data from 5 cells. Right panels: representative monovalent whole-cell $I_{Kir7.1}$ recordings from transiently transfected HEK293 cells in response to a voltage ramp as indicated and exposed to varying concentrations of P4. Dotted lines represent baseline.

Figure S4

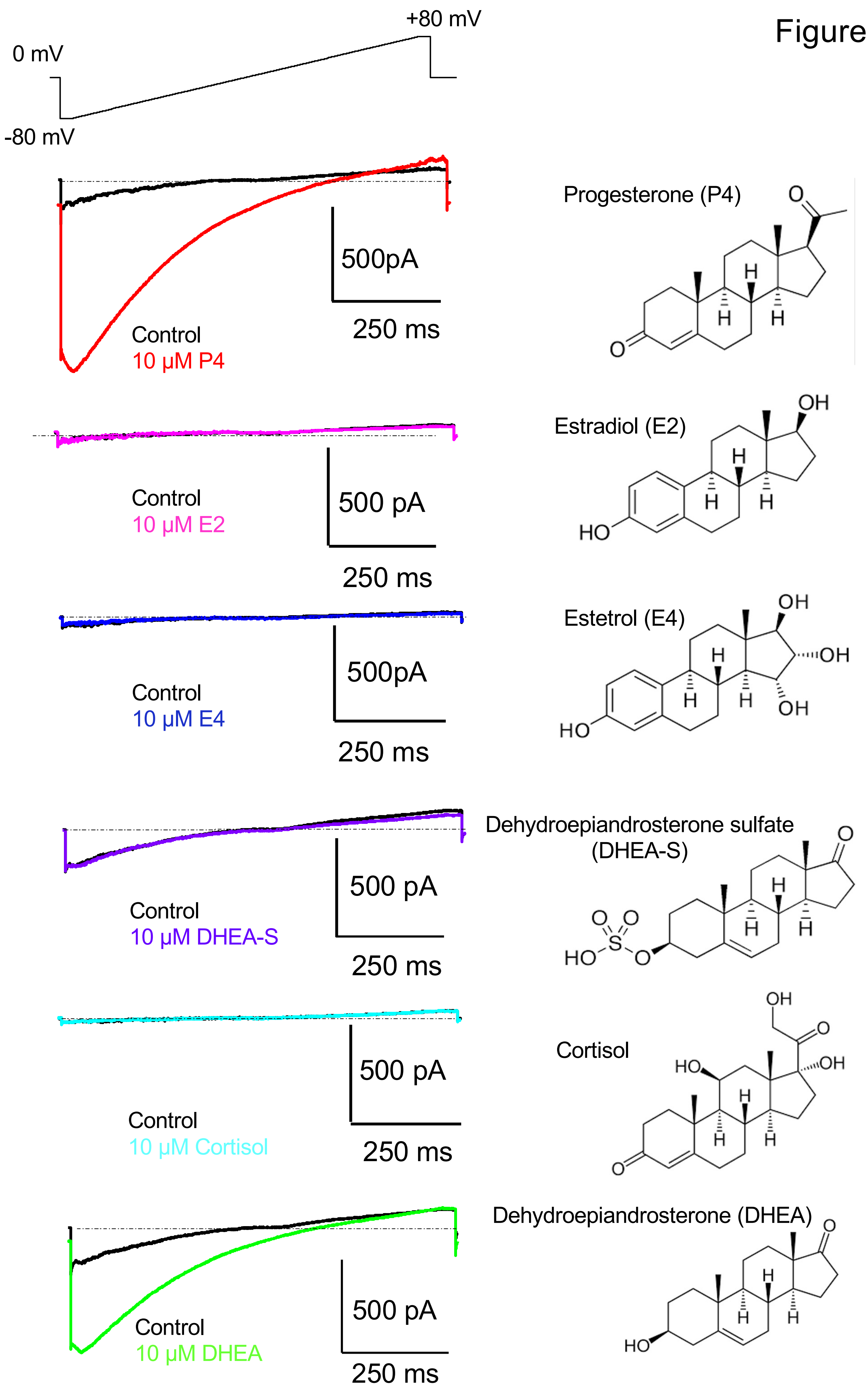
+80 mV
0 mV
-80 mV
Progesterone (P4)
500pA
250 ms
Control
10 μM P4
Estradiol (E2)
500 pA
250 ms
Control
10 μM E2
Estetrol (E4)
500pA
250 ms
Control
10 μM E4
Dehydroepiandrosterone sulfate (DHEA-S)
500 pA
250 ms
Control
10 μM DHEA-S
Cortisol
500 pA
250 ms
Control
10 μM Cortisol
Dehydroepiandrosterone (DHEA)
500 pA
250 ms
Control
10 μM DHEA

Supplemental Figure 4. Potentiation of hKir7.1 by endogenous steroids. Representative traces recorded from HEK293 cells transfected with pIRES2-EGFP-hK$_{ir}$7.1 construct as described above show different responses to the corresponding steroids. Right panels depict their corresponding chemical structures.

Figure S5

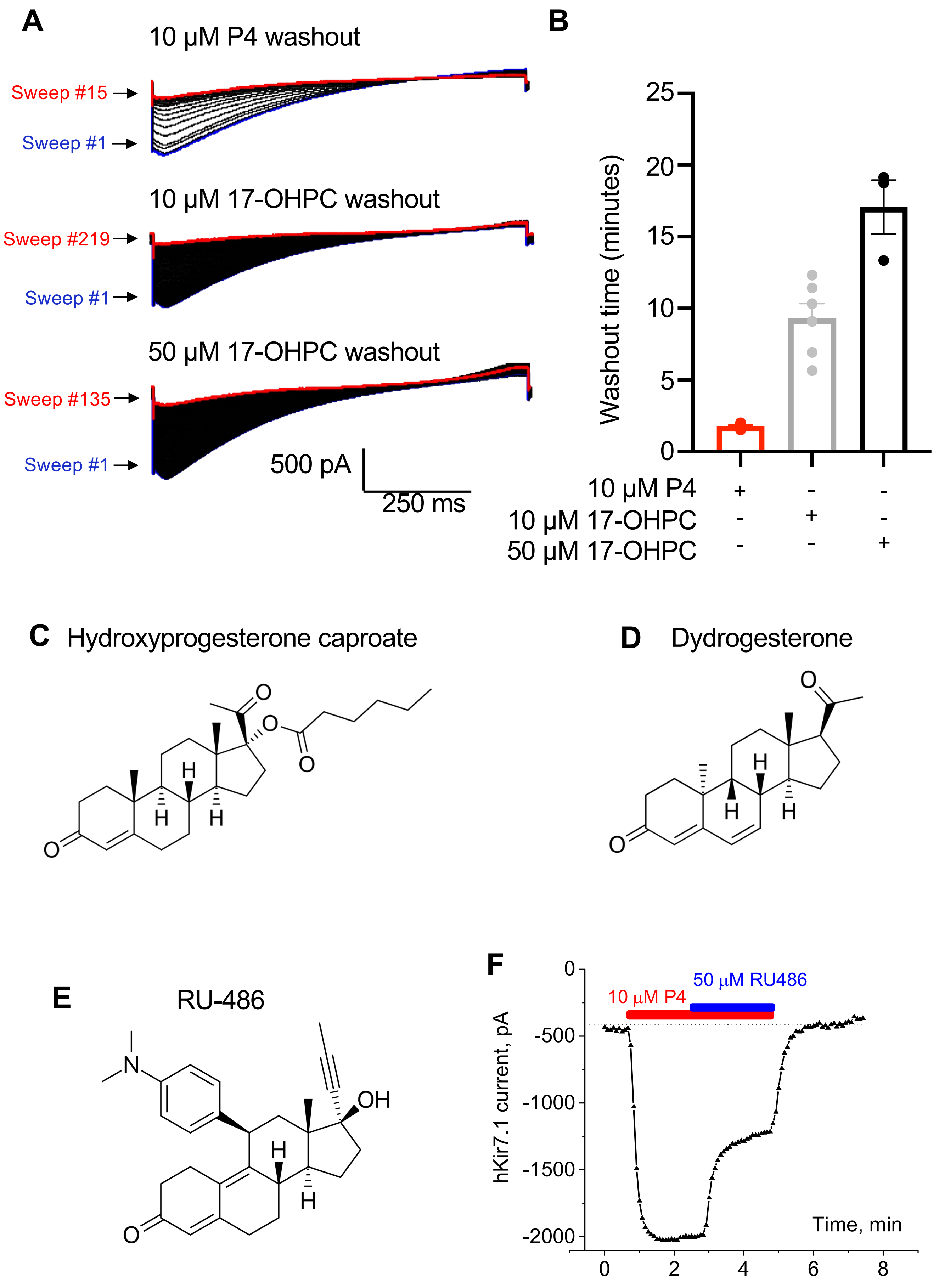

A
10 µM P4 washout
Sweep #15
Sweep #1
10 µM 17-OHPC washout
Sweep #219
Sweep #1
50 µM 17-OHPC washout
Sweep #135
Sweep #1
500 pA
250 ms
B
Washout time (minutes)
0
5
10
15
20
25
10 µM P4 + - -
10 µM 17-OHPC - + -
50 µM 17-OHPC - - +
C Hydroxyprogesterone caproate
D Dydrogesterone
E RU-486
F
10 µM P4
50 µM RU486
hKir7.1 current, pA
0
-500
-1000
-1500
-2000
Time, min
0
2
4
6
8

Supplemental Figure 5. Effect of synthetic steroids on recombinant hKir7.1.
A. Representative washout traces recorded from HEK293 cells expressing hKir7.1 following stimulation with either 10 µM P4, 10 µM 17-OHPC, or 50 µM 17-OHPC. Sweep #1 in blue shows the last recording in the solution with the corresponding steroid after reaching the maximal potentiation by the corresponding compound, and immediately before washout was initiated. The sweep in red indicates the last recording in the washout solution when the amplitude failed to decrease further and has reached steady-state. **B.** Washout times required to reach full steady-state were plotted in minutes against the corresponding steroids. Statistics are as follows: (P4): 1.77 ± 0.06, n=8; (10 µM 17-OHPC): 9.29 ± 1.06, n=6; (50 µM 17-OHPC): 17.08 ± 1.87, n=3. **C-E.** Chemical structures of the synthetic steroids **F.** Development of hKir7.1 inward current at -80 mV upon P4 activation and subsequent inhibition by RU486.

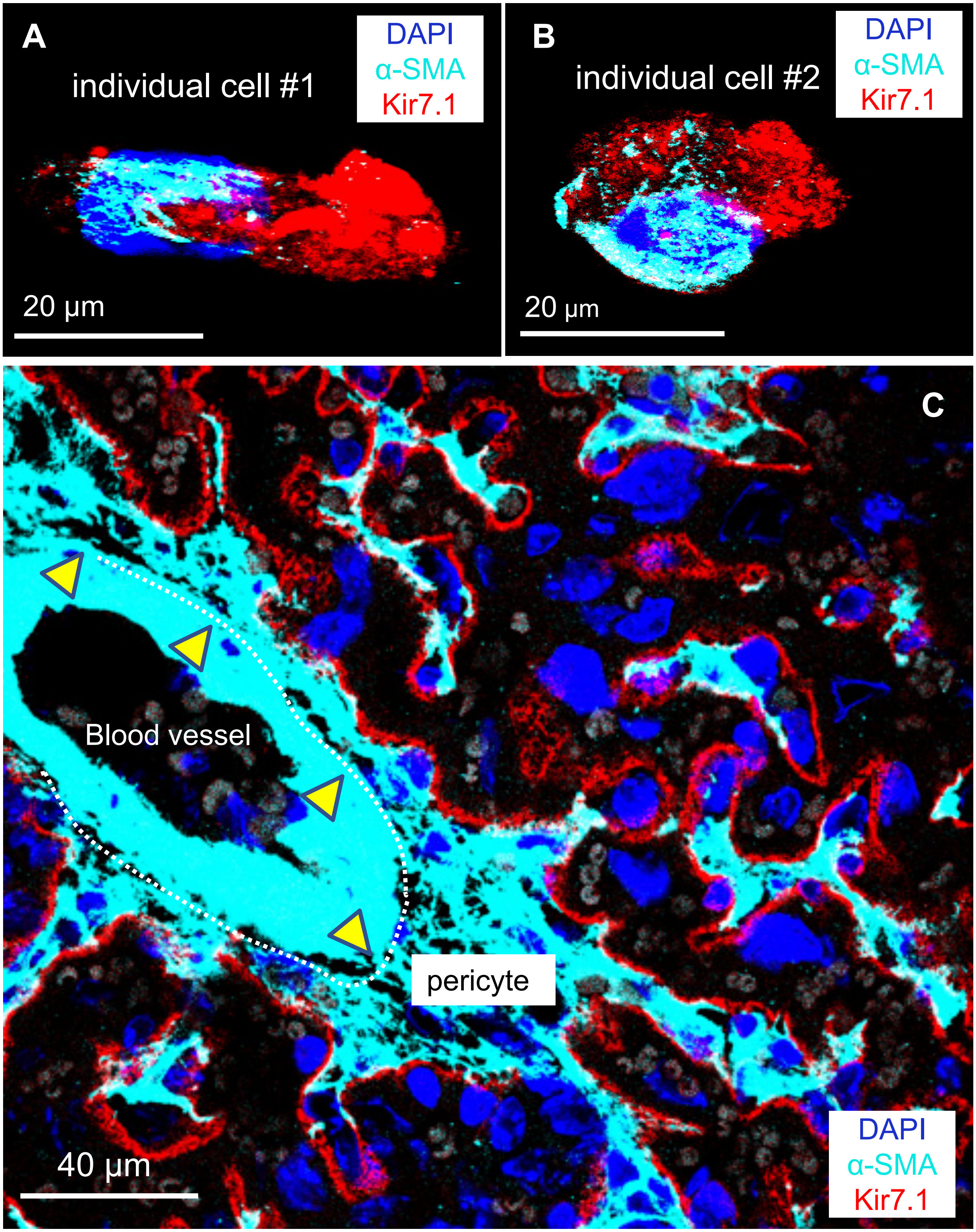
A
individual cell #1
DAPI
α-SMA
Kir7.1
20 μm
B
individual cell #2
DAPI
α-SMA
Kir7.1
20 μm
C
Blood vessel
pericyte
40 μm
DAPI
α-SMA
Kir7.1

Figure S6

Supplemental Figure 6. Kir7.1 is asymmetrically expressed in murine placental pericytes. A-B. Two representative immunostaining of isolated pericytes from 15.5 dpc placenta stained with anti-Kir7.1 (red) and α-SMA (cyan) antibodies. The ion channel is asymmetrically distributed on the cellular membrane. Cell nuclei are stained with DAPI (blue). **C.** Zoomed in segment from Figure 6E shows Kir7.1-expressing (red) placental pericytes forming tight connections with smooth muscles of the blood vessel. The apical sides of the smooth-muscle-adjacent pericytes are indicated by yellow arrowheads.

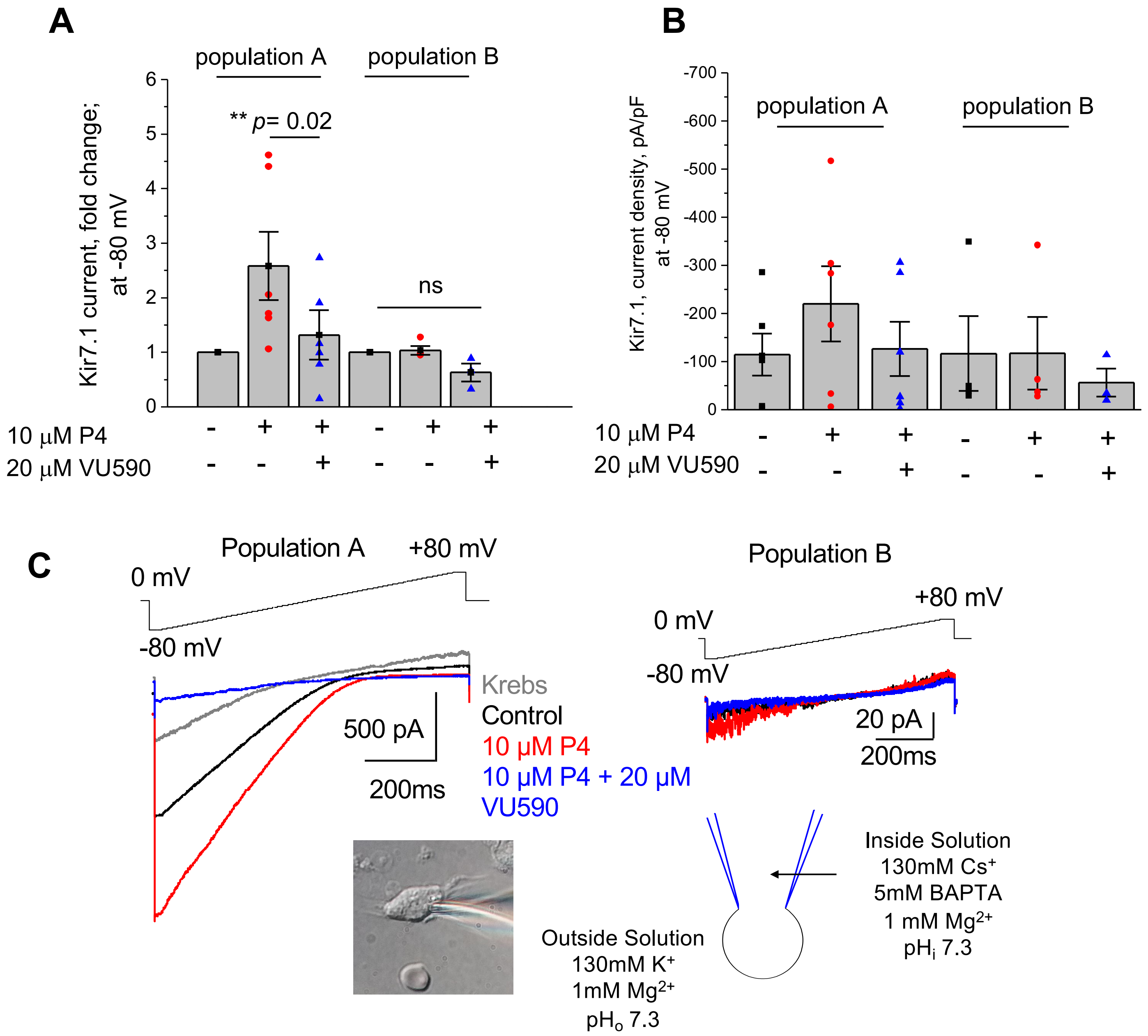
A
population A
population B
** p= 0.02
ns
Kir7.1 current, fold change; at -80 mV
10 µM P4
20 µM VU590
B
population A
population B
Kir7.1, current density, pA/pF at -80 mV
10 µM P4
20 µM VU590
C
Population A
0 mV
+80 mV
-80 mV
Krebs
Control
10 µM P4
10 µM P4 + 20 µM VU590
500 pA
200ms
Population B
0 mV
+80 mV
-80 mV
20 pA
200ms
Outside Solution
130mM K+
1mM Mg2+
pHo 7.3
Inside Solution
130mM Cs+
5mM BAPTA
1 mM Mg2+
pHi 7.3

Figure S7

Supplemental Figure 7. Kir7.1 recordings from isolated murine placental pericytes. A-B. The averaged fold increase (A) and current densities (B) of mouse Kir7.1 recorded at -80 mV from two different populations of placental pericytes: population A that responded to P4 and population B that showed weak P4 response. **A.** Averaged fold increase, normalized to control, n=6. Statistics for population A are as follows: (P4): 2.58 ± 0.63, n=6; (P4+VU590): 1.32 ± 0.45, n=6; statistics for population B are as follows: (P4): 1.04 ± 0.08, n=4; (P4+VU590): 0.63 ± 0.16, n=3. **B.** Averaged current densities. Statistics for population A are as follows: (control): -114.18 ± 43.49, n=6; (P4): -220.09 ± 77.87, n=6; (P4+VU590): -126.10 ± 56.49, n=6. Statistics for Population B are as follows: (control): -116.57 ± 77.6, n=4; (P4): -117.37 ± 75.37, n=4; (P4+VU590): -56.11 ± 29.45, n=3. Statistical significance was calculated using the paired t-test, and the significance of changes is indicated as follows: ** $p \leq 0.05$; data are means ± SEM.
C. Corresponding representative traces from 15.5 dpc placental pericytes show variable Kir7.1 activity (populations A and B) that respond to P4 stimulation. P4-activated currents were potently inhibited by the Kir7.1-specific antagonist VU590 further confirming Kir7.1 expression in placental pericytes.

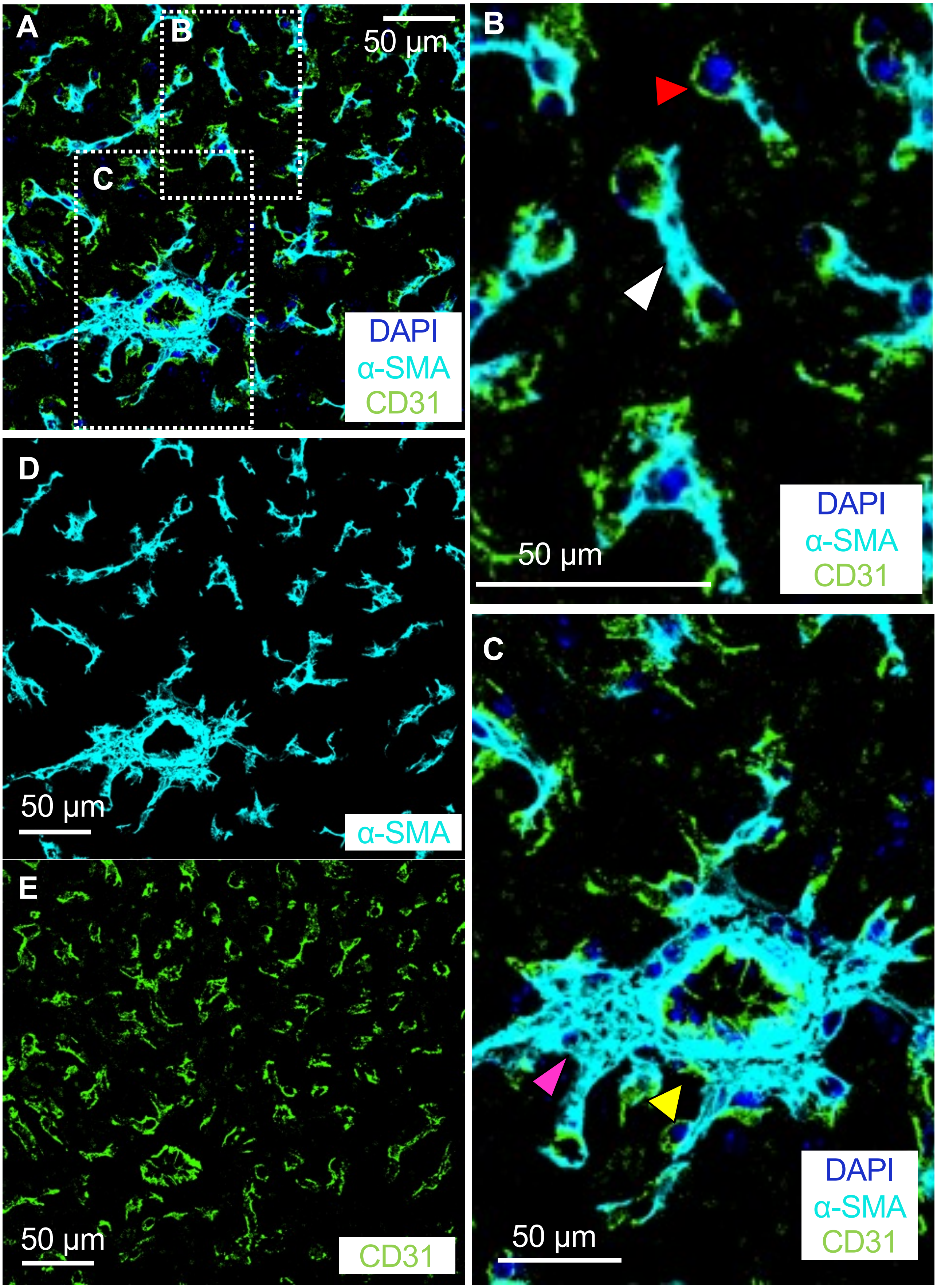
A
B
C
50 µm
DAPI
α-SMA
CD31
B
50 µm
DAPI
α-SMA
CD31
D
50 µm
α-SMA
E
50 µm
CD31
C
50 µm
DAPI
α-SMA
CD31

Figure S8

Supplemental Figure 8. Interaction between endothelial cell, pericytes and vasculature in murine placental labyrinth. A. Horizontal cross-section through the labyrinth at 15.5 dpc as described in Figure 6A, plane (c). Placental pericytes are visualized by their characteristic pleomorphic morphology and intense α-SMA (cyan) staining, while capillaries are visualized by their specific endothelial marker CD31. **B.** Zoomed in section of (A) with pericytes indicated by white arrowhead connecting capillaries indicated by red arrow. **C.** Zoomed in section of (A) with combined α-SMA (cyan) and CD31 (green) staining shows pericytes (pink arrowhead) and smooth muscle vasculature surrounding blood vessel (yellow arrowhead). **D-E.** Single channel panels of (A).

Figure S9

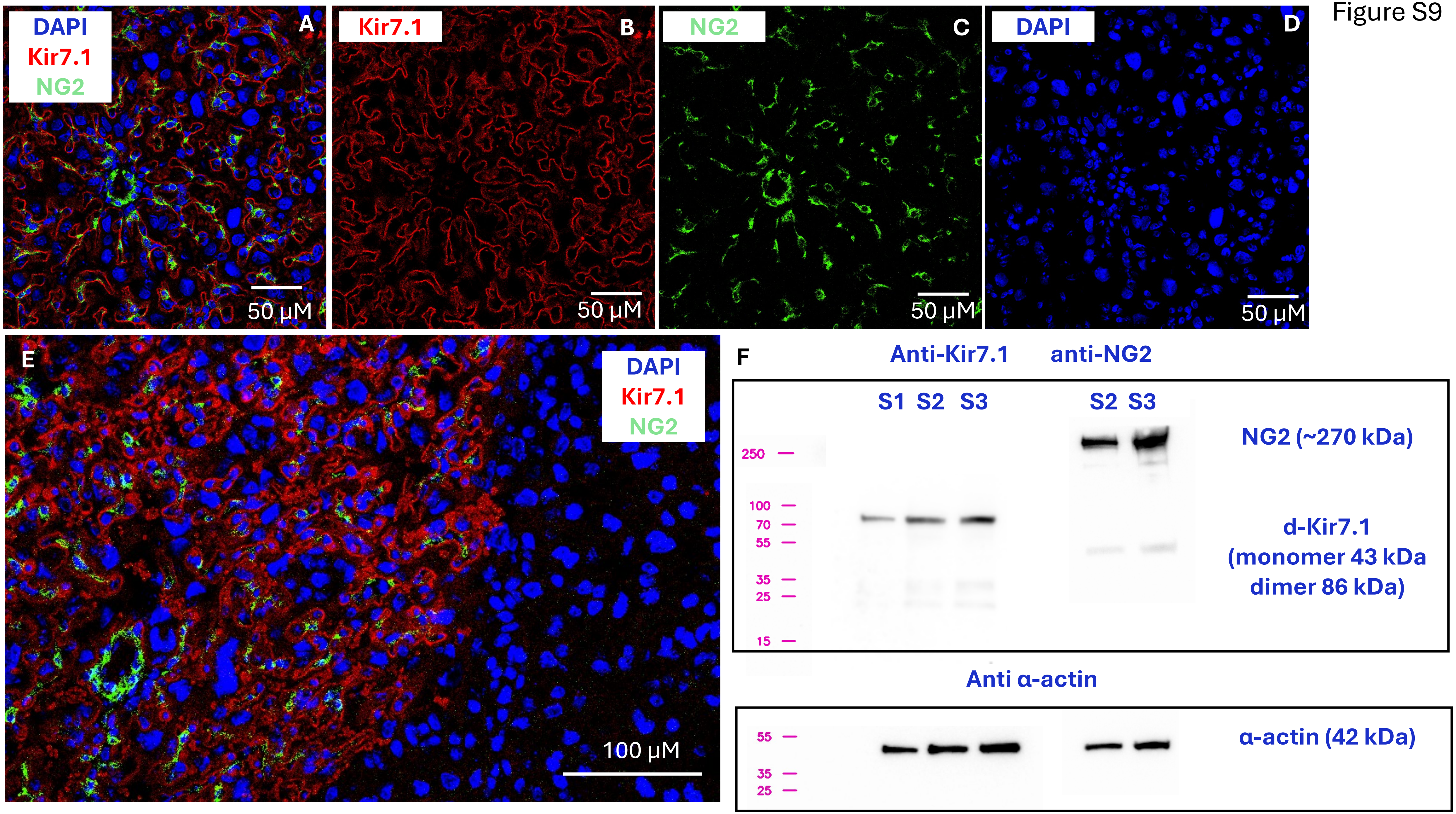
A
DAPI
Kir7.1
NG2
50 μM
B
Kir7.1
50 μM
C
NG2
50 μM
D
DAPI
50 μM
E
DAPI
Kir7.1
NG2
100 μM
F
Anti-Kir7.1
anti-NG2
S1 S2 S3
S2 S3
250
100
70
55
35
25
15
NG2 (~270 kDa)
d-Kir7.1
(monomer 43 kDa
dimer 86 kDa)
Anti α-actin
55
35
25
α-actin (42 kDa)

Supplemental Figure 9. Detection of Kir7.1 in murine and human placentae. A-E. Horizontal cross-sections through murine labyrinth at 15.5 dpc as described above. **A.** Zoomed in portion of murine placental labyrinth co-stained with anti-Kir7.1, anti-NG2 and DAPI with all three channels superimposed. Red and green signals confirm that Kir7.1 is expressed in pericytes, while absent from vascular smooth muscles. Cell nuclei are stained by DAPI (blue). **B-D.** Single channel panels of (A). **B**. Strong Kir7.1 staining (red) with typical membrane localization of the ion channel. **C.** The same section as in (A-B) visualized with anti- NG2 (green) antibodies reveals cells with pleomorphic morphology typical for placental pericytes. In addition to pericytes, NG2 is expressed in mesenchymal stem cells, macrophages, and vascular smooth muscles. The latter can be observed in (A), (C) and (E) as a circular region of Kir7.1-less group of cells indicating a blood vessel. Membrane-localized Kir.7.1 signal (red) was co-localized with larger portion of NG2-containing cells (green). **D.** Cell nuclei are stained by DAPI (blue). **E.** A zoomed-out region of murine labyrinth co-stained as described in (A) shows a border between Kir7.1-enriched labyrinth layer (red) and spongiotrophoblast layer (right) that lacks pericytes. **F.** Western blot of the protein lysates isolated from three regions from human postpartum placenta (S1, S2, S3) obtained from Discovery Life Sciences (DLS.com) tissue biobank. The tissue was collected at 38 weeks gestation. The blots were visualized with the same anti-Kir7.1 and anti-NG2 monoclonal antibodies. In the placenta, hKir7.1 exists as a dimer (86kDa for the dimer vs. its typical monomeric 43kDa isoform). Anti-alpha-actin was used as a loading control (lower panels).

Video S1. Myometrial contractions recorded under control conditions. Time-lapse brightfield macroscopic recordings of myometrial contractions in acute uterine tissue sections (300 µm; longitudinal cutting plane). Both circular and longitudinal muscle fibers are visible. Video shows two consecutive 10-min recordings, interrupted by a 10-min ‘wash-in’ period. Here, control conditions were maintained (i.e., sections were constantly superfused with $\mathbf{S_2}$ (RT)) throughout the recording. Original trace (black; pixel displacement *versus* time) displaying representative myometrial contractions in a manually defined ROI. Alternating positive and negative peaks correspond to individual contraction-relaxation events. 2 Hz frame rate; 40 x video replay speed. Note that the tissue maintains pronounced contractile motion throughout the entire recording.

Video S2. P4-dependent reduction in myometrial contraction strength. Time-lapse brightfield macroscopic recordings of myometrial contractions in acute uterine tissue sections (300 µm; cutting plane along the circular muscle fibers). Tissue / muscle fiber orientation as indicated. Video shows two consecutive 10-min recordings, interrupted by a 10-min 'wash-in' period, during which the tissue is incubated in P4 (30 µM). Original trace (black (control) / red (P4 treatment); pixel displacement *versus* time) shows representative myometrial contractions in a manually defined ROI. Alternating positive and negative peaks correspond to individual contraction-relaxation events. 2 Hz frame rate; 40 x video replay speed.

Video S3. Increase in myometrial contractility upon VU590 (+ P4) treatment. Time-lapse brightfield macroscopic recordings of myometrial contractions in acute uterine tissue sections (300 µm; cutting plane along the circular muscle fibers). Tissue / muscle fiber orientation as indicated. Video shows two consecutive 10-min recordings, interrupted by a 'wash-in' period, during which the tissue is exposed to both VU590 (100 µM) and P4 (30 µM). Original trace (black (control) / blue (VU590 + P4 treatment); pixel displacement *versus* time) shows representative myometrial contractions in a manually defined ROI. Alternating positive and negative peaks correspond to individual contraction-relaxation events. 2 Hz frame rate; 40 x video replay speed.
